# Fe^2+^‐ Induced Activation of Single and Dual Metal Site—Lattice Oxygen Mechanism in Fe Rich NiFe‐LDHs for Oxygen Evolution Reaction

**DOI:** 10.1002/advs.202521259

**Published:** 2026-03-04

**Authors:** Nithinraj Panangattu Dharmarajan, Mohammed Fawaz, Vanshree Parey, Sudip Chakraborty, CI Sathish, Thi Thuy Kieu Tran, Xuan Minh Chau Ta, Siddulu Naidu Talapaneni, Zhixuan Li, Kavitha Ramadass, Antonio Tricoli, Jae‐Hun Yang, Ajayan Vinu

**Affiliations:** ^1^ Global Innovative Centre of Advanced Nanomaterials College of Engineering, Science and Environment University of Newcastle Callaghan NSW Australia; ^2^ Nanotechnology Research Laboratory Faculty of Engineering University of Sydney Sydney NSW Australia; ^3^ Materials Theory For Energy Scavenging (MATES) Lab Harish‐Chandra Research Institute (HRI), Allahabad HBNI, Jhunsi Prayagraj (Allahabad) India

**Keywords:** lattice oxygen mechanism, layered double hydroxide, metal–organic framework, oxygen defects, oxygen evolution reaction

## Abstract

Developing a durable and efficient oxygen evolution reaction (OER) catalyst without noble metals is essential for the economical and sustainable production of hydrogen by alkaline water electrolysis. Recent studies have advocated triggering the lattice oxygen mechanism (LOM) to reduce the overpotential of catalysis and overcome the inherent limitations of the adsorbate evolution mechanism (AEM). Herein, we explored a facile and scalable method for synthesizing an iron‐rich nickel‐layered double hydroxide (NiFe‐LDH) with a unique hollow nanocapsule morphology using a metal‐organic framework (MOF) MIL‐88A as a sacrificial template for OER. The transformation of the MOF into hollow NiFe‐LDH provides abundant lattice oxygen defects and alters the Fermi level of O 2p, triggering LOM. As a result, the material exhibited outstanding OER performance, with a low overpotential of 244 mV at 10 mAcm^−2^, and excellent long‐term durability. Ex‐situ X‐ray Photoelectron Spectroscopy (XPS) analysis of the catalyst before and after the OER reaction revealed a high density of surface oxygen defects in the Fe‐rich NiFe‐LDH, which is a prerequisite for LOM activation. Also, through in‐situ Raman spectroscopy, which monitored the appearance of superoxide intermediates (1040–1250 cm^−1^), revealed strong evidence for the involvement of the single metal site LOM and dual metal site LOM pathways in the OER. The presence of Fe^2+^ and Fe^3+^ in Fe‐rich NiFe‐LDH and the possible LOM mechanism were further evaluated using computational analyses.

## Graphical abstract

Fe‐rich NiFe‐LDH catalysts derived from MOFs, showing superior OER activity via lattice oxygen mechanism activation.

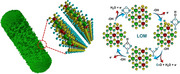

## Introduction

The escalating energy crisis and the impending threats of climate change, exacerbated by human activities, have intensified the urgent need for clean, efficient, and economically viable alternative energy sources [1]. Among the sustainable energy sources, green hydrogen production has garnered global prominence in recent years [2]. Electrochemical water splitting, a process that utilizes electricity to split water into hydrogen and oxygen, has attracted significant global attention as a promising future energy source. This process hinges on the simultaneous occurrence of two half‐cell reactions: the cathodic hydrogen evolution reaction (HER) and the anodic oxygen evolution reaction (OER) [3, 4, 5, 6, 7, 8]. Although HER proceeds efficiently, the OER represents a critical bottleneck due to its sluggish 4‐electron pathway and the transfer of protons and intermediates in the adsorbate evolution mechanism (AEM) pathway with high theoretical free energy requirements, resulting in a high overpotential [9]. Recent studies have explored an alternative lattice oxygen mechanism (LOM) pathway that utilizes lattice oxygen (LO) sites and oxygen vacancies as active sites exhibiting reduced theoretical overpotential requirements compared to that of the AEM pathway at high‐valent metal active sites. However, the lack of understanding of the LOM mechanism and how to trigger such reactions in potential catalyst materials limits the large‐scale industrial implementation of this technology. To address these challenges, the development of low‐cost and highly stable electrocatalysts with minimal overpotentials through the activated LOM pathway is essential for further progress in this field. Earth‐abundant transition metal‐based (Fe, Ni, Co, etc.) electrocatalysts, such as transition metal oxides, oxyhydroxides, layered double hydroxides (LDHs), oxyphosphates, perovskites, and high‐entropy materials, have emerged as promising candidates. Among them, LDH nanostructures with the unique 2D structure, composed of positively charged metal hydroxide layers and charge‐compensation anions, can provide abundant exposed LO active sites, making them ideal candidates for LO activation for OER applications.

Among the various LDHs materials, NiFe‐based LDHs have emerged as promising anode materials for alkaline water electrolysis, attracting considerable attention in recent years. Corrigan et al. demonstrated the significant influence of Fe impurities on the OER overpotential in a NiO thin film under alkaline conditions, which empowered the development of various NiFe‐based LDH catalysts with enhanced OER activity [10]. Despite their unequivocal substantial enhancement in the OER activity [11, 12, 13], the precise nature of OER active sites in NiFe‐based LDH materials remains a topic of ongoing debate [14, 15, 16, 17, 18, 19]. While the synergistic combination and interactions of Ni and Fe sites have been widely acknowledged as crucial factors in improving OER performance, recent studies have proposed alternative OER mechanisms involving LO sites instead of metal active sites [20, 21]. The exact reaction pathway of these materials remains a subject of active research and discussion among researchers. Recently, MOF‐derived LDHs nanostructured materials have offered a promising approach to develop a high‐performing catalyst by simultaneously integrating high surface area, excellent electronic conductivity, and accessible active sites, which facilitated the way for achieving enhanced catalytic efficiency [22, 23, 24, 25]. Additionally, compared to single‐metal‐based catalysts, bimetallic catalysts exhibit exceptional performance owing to their synergetic effects, efficient charge transfer, and the ability to interact with reactants while stabilizing the active metal sites against corrosion and dissolution [26, 27]. This combination of attributes makes NiFe‐based LDH materials highly attractive for LO assisted alkaline water electrolysis applications.

Herein, we demonstrate a facile MOF‐template‐assisted strategy for synthesizing Fe‐rich NiFe‐LDHs with uniform hollow nanoarchitectures for efficient electrocatalytic oxygen generation. The developed catalyst exhibits exceptional performance, including a low overpotential, low Tafel slope, high turnover frequency, large electrochemically active surface area, and extended catalyst stability, underscoring its superior efficacy in the OER. ICP‐MS analysis of the electrolyte collected at periodic intervals during the stability test indicates negligible metal leaching, underscoring the electrochemical stability of the catalyst. We employed near‐edge X‐ray absorption fine structure (NEXAFS) and X‐ray photoelectron spectroscopy (XPS) to gain insight into the local coordination and oxidation states of the transition metals and to understand the structure and defect sites of the electrocatalysts. A comprehensive ex‐situ XPS study revealed a mixed valence state of Fe^2+^/Fe^3+^ in Fe‐rich NiFe‐LDH and abundant oxygen vacancy defect sites, which can promote the LOM pathway. Theoretical calculations further confirmed the presence of Fe^2+^ in the Fe‐rich NiFe‐LDH (Fe >25%), and free energy calculations indicate a lower OER overpotential on LO coordinated with Fe^2+^ compared to Fe^3+^. Furthermore, in‐situ Raman spectroscopy was employed to monitor the formation of superoxide (O─O^−^, O─O^2−^) under different applied potentials and confirms the single metal site LOM and dual metal site LOM pathways in the Fe‐rich NiFe‐LDH catalyst.

## 1 Results and Discussion

### 1.1 Synthesis of Hollow Fe‐Rich NiFe‐LDH and Elucidating Its Structure, Morphology and Characterization

**FIGURE 1:**
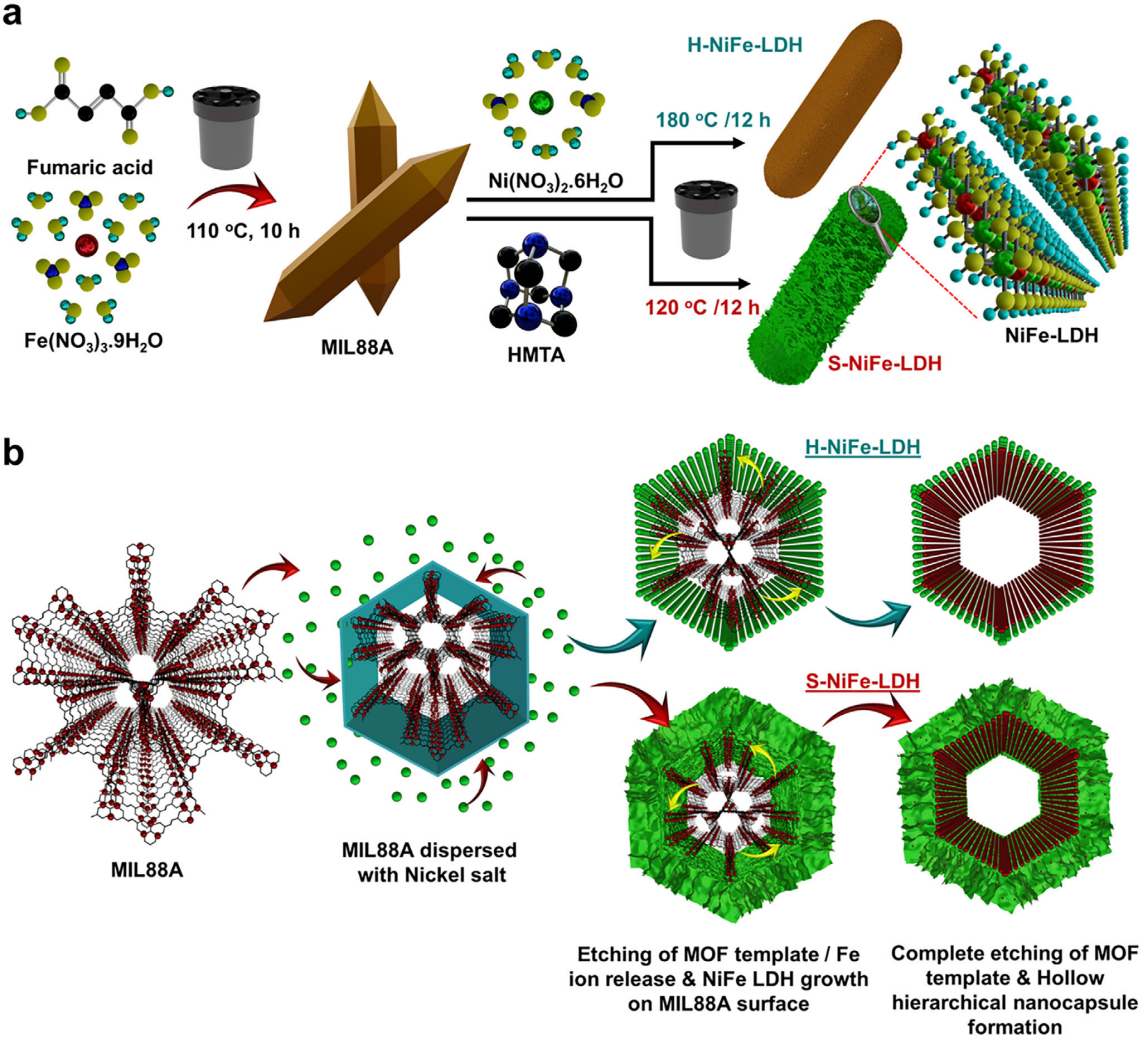
Schematic illustration of the (a) synthesis pathway and (b) LDH formation via hollow etching method for H‐NiFe‐LDH and S‐NiFe‐LDH.

The fabrication and mechanism involved in constructing the Fe‐rich NiFe‐LDH are schematically represented in Figure 1. MIL88A was synthesized hydrothermally at 110°C, and the prepared sample showed an average length of ∼ 3.3 µm and a width of ∼ 400 nm (Figure S1a,b) [28]. The well‐defined hierarchical NiFe‐LDH hollow nanocapsules were synthesized via further hydrothermal treatment of MIL88A with various concentrations of Ni^2+^ and hexamethylenetetramine (HMTA) at defined temperatures, where MIL88A acted as the Fe source and sacrificial morphology template. By varying the synthesis temperature and the amount of Ni precursor and HMTA, different morphologies of NiFe‐LDH (Figure 1a) were fabricated. The NiFe‐LDH material prepared at 180°C under the hydrothermal conditions with the NiNO_3_.6H_2_O and HMTA displayed a hollow nanocapsule‐like NiFe‐LDH (H‐NiFe‐LDH), whereas sheet‐encapsulated hollow NiFe‐LDH (S‐NiFe‐LDH) was formed at 120°C (Figure 1a). A detailed description of the synthesis procedure is provided in the Supporting Information. During the hydrothermal process, HMTA acts as a slow and controlled hydroxide‐releasing agent, facilitating the controlled nucleation and growth of NiFe‐LDH on the MIL88A surface. Under harsh hydrothermal conditions, additional hydroxide ions are generated from both HMTA and water, which promotes the dissolution of the Fe species from the inner core of MIL88A particles [29]. The released Fe ions with Ni ions in the presence of hydroxide form NiFe LDH structures on the MIL88A shell. At a high temperature (180°C), the MIL88A core was gradually etched away, leaving a hollow void inside the particles, facilitating the hierarchical nanocapsule‐like hollow morphology of the Fe‐rich NiFe‐LDH (Figure 1b). However, at a lower temperature (120°C), the abundant Ni^2+^ ions and dissolved Fe^3+^ ions formed LDH sheets on the surface of the MOF particle, resulting in a sheet‐encapsulated hollow morphology. The residual fumaric acid ligands and excess metal ions were completely removed through multiple washing steps with water and ethanol.

**FIGURE 2:**
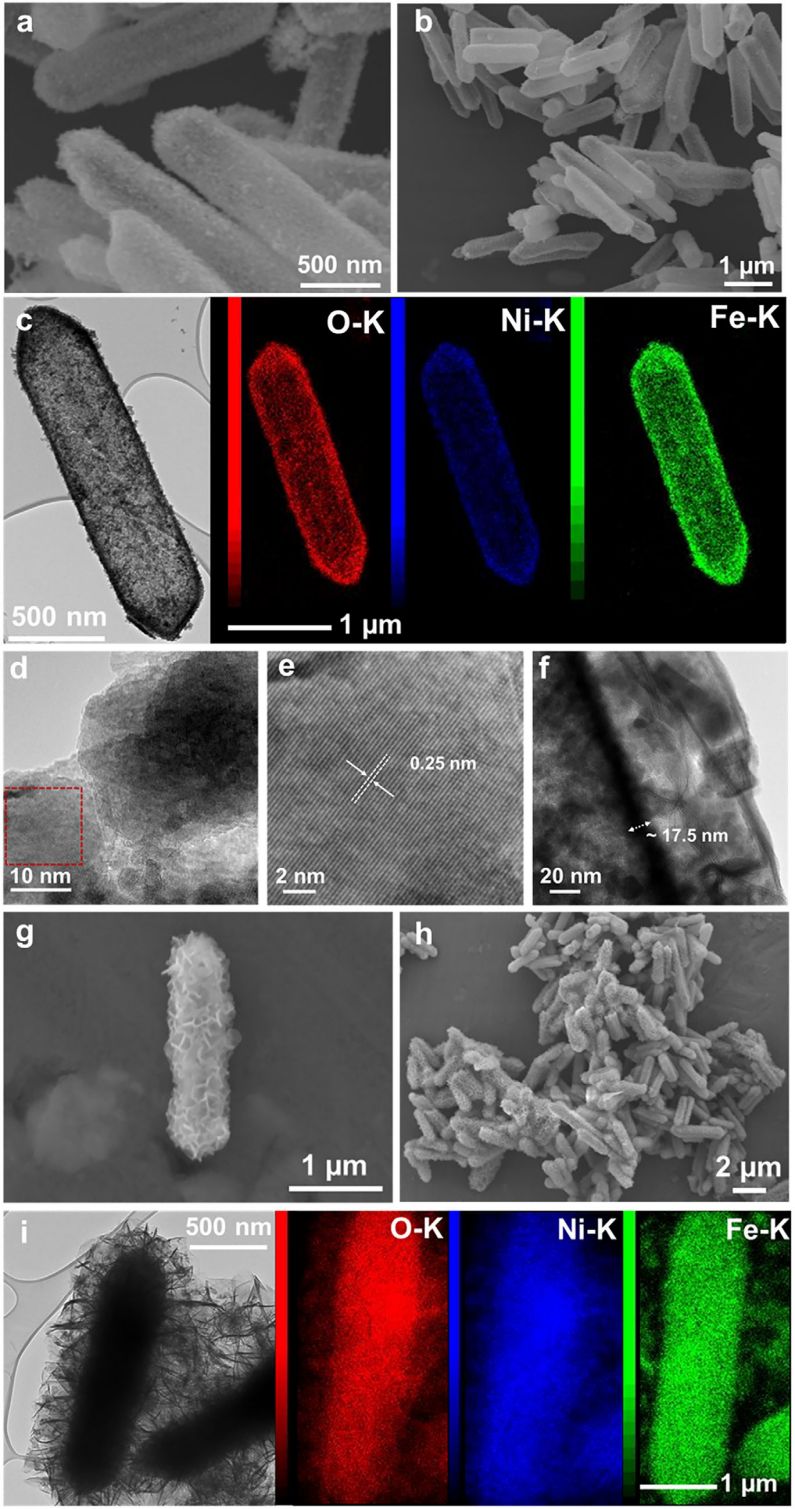
(a,b) FESEM images at higher and lower magnifications, (c–f) HR‐TEM image and elemental mapping showing the distribution of Ni, Fe, and O atoms of H‐NiFe‐LDH, (e) enlarged image of (d) displaying lattice fringes, (f) TEM image displaying the wall thickness between hollow and outer surface, (g, h) FESEM and (i) TEM image and elemental mapping of Ni, Fe, and O atoms of S‐NiFe‐LDH.

Field‐emission scanning electron microscopy (FESEM) images of H‐NiFe‐LDH demonstrate a rough surface with a well‐preserved, uniform capsule‐like morphology with an average length of approximately 3.5 µm and a thickness of 500 nm, which is slightly larger in dimensions than the MIL88A MOF (Figure 2a,b), indicating that small and thin LDH particles grew on the surface of the MOF particles. In contrast, the high‐magnification FESEM images of S‐NiFe‐LDH show sheet‐like morphologies vertically grown on the surface of the MOF particles (Figure 2g,h). Furthermore, transmission electron microscopy (TEM) analysis revealed that the H‐NiFe‐LDH exhibited a hollow morphology (Figure 2c,d) with a wall thickness of ∼18 nm (Figure 2f). The distinct contrast between the lamellar shell and the visible central hollow space clearly shows the internal cavity of the NiFe‐LDH nanocapsules. It should be noted that the controlled amount of HMTA in the synthesis plays a crucial role in the formation of the hollow cavity and growth of the nanocapsules. The TEM elemental mapping images confirmed the homogeneous distribution of Ni, Fe, and O atoms throughout the H‐NiFe‐LDH surface. The observed lattice fringes with a spacing of 0.25 nm (Figure 2e) correspond to the (012) planes of NiFe‐LDH [30], which further confirms the formation of the LDH structure. The TEM images of the S‐NiFe‐LDH also exhibit a hollow structure, but a wrinkled sheet‐like growth on the top can be discerned (Figure 2i). The elemental mapping images show a homogeneous distribution of Ni and O atoms, whereas Fe atoms are more concentrated in the core of the S‐NiFe‐LDH structure. The Fe‐to‐Ni composition, determined by inductively coupled plasma optical emission spectroscopy (ICP‐OES), was determined to be Ni_0.61_Fe_0.39_ and Ni_0.68_Fe_0.32_ for H‐NiFe‐LDH and S‐NiFe‐LDH, respectively, where the molar ratio of Ni to Fe is less than 2, confirming the presence of Fe‐rich NiFe‐LDH (ESI). To confirm the formation of NiFe‐LDH instead of the Ni‐LDH/Fe‐LDH mixture, we conducted an elemental analysis of the localized area (Figure S2). The even distribution of Ni and Fe atoms on the surface of H‐NiFe‐LDH indicates that the material is highly unlikely to be a physical mixture of separate Ni‐LDH and Fe‐LDH phases. The surface areas and average pore sizes of S‐NiFe‐LDH and H‐NiFe‐LDH were determined using N_2_ adsorption‐desorption analysis (Figure S3a). Electronic Supporting Information (ESI)). The synthesized materials exhibited high specific surface areas of 126.5 m^2^g^−1^ for H‐NiFe‐LDH and 170.8 m^2^g^−1^ for S‐NiFe‐LDH due to the hollow structure. As evident from the TEM images of S‐NiFe‐LDH, the ultrathin LDH sheets grown on the hollow surface contributed to a higher specific surface area than that of H‐NiFe‐LDH. Likewise, the mesopores in the samples (Figure S3b) are highly beneficial for more active sites, electrolyte transfer, and rapid mass transfer for the OER.

**FIGURE 3:**
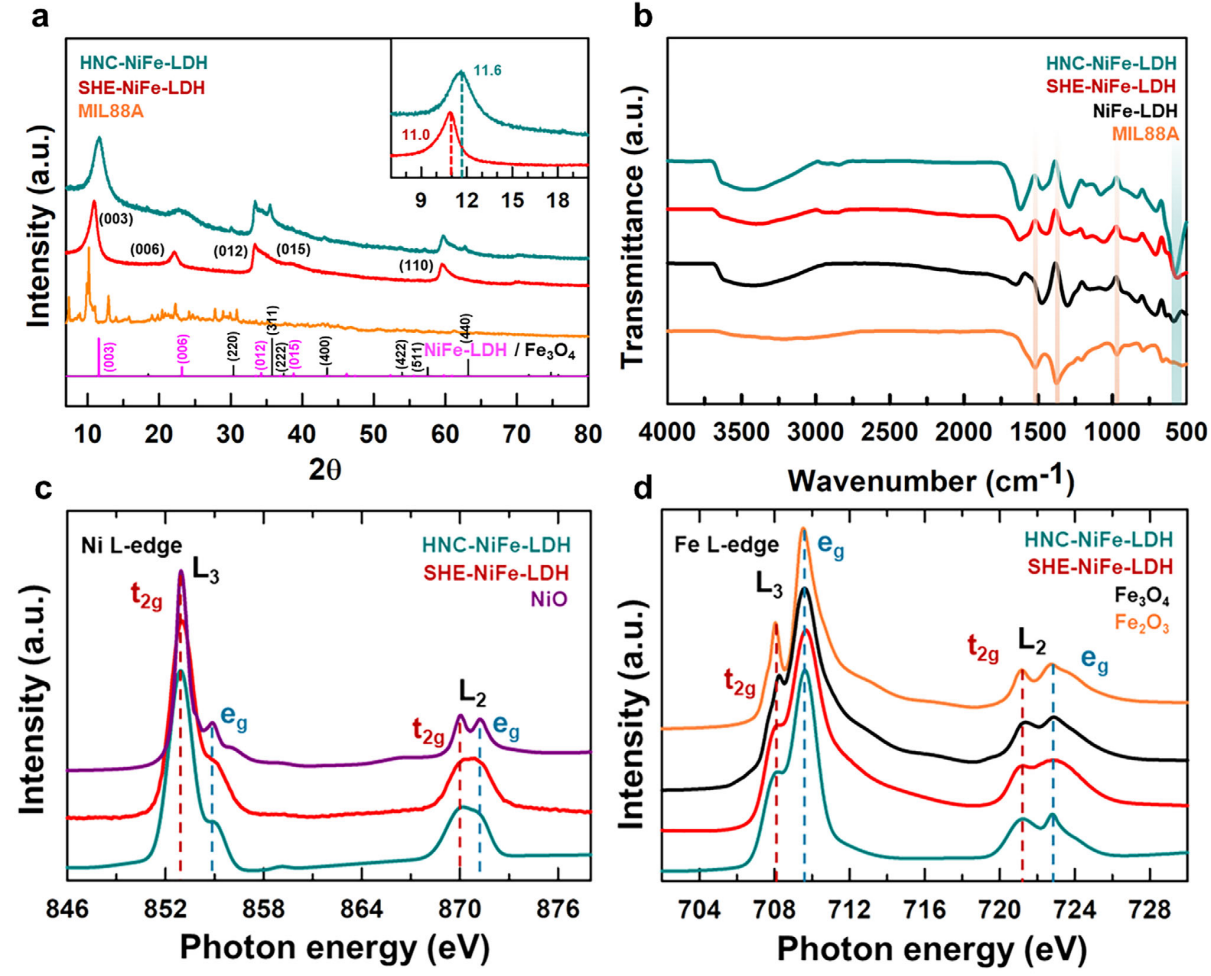
(a) XRD patterns of MIL88A, S‐NiFe‐LDH, H‐NiFe‐LDH and references (NiFe‐LDH and Fe_3_O_4_), (b) FTIR spectra of MIL88A, S‐NiFe‐LDH, H‐NiFe‐LDH, and carbonate‐intercalated NiFe‐LDH as a reference. NEXAFS spectra (c) Ni L‐edge and (d) Fe L‐edge of S‐NiFe‐LDH, H‐NiFe‐LDH, along with NiO, Fe_3_O_4_ and Fe_2_O_3_ as references.

The X‐ray diffraction (XRD) patterns of the H‐NiFe‐LDH and S‐NiFe‐LDH (Figure 3a) demonstrated a typical hydrotalcite‐like structure revealing the formation of layered double hydroxides [31]. The observed XRD peaks exhibited relatively broadened profiles, suggesting a small crystallite size, and additionally, the asymmetrically broadened peak of (012) at ∼ 33.5° represents the presence of turbostatic layer stacking [32, 33]. For S‐NiFe‐LDH, the XRD peaks corresponding to the (003), (006), (012), (015), and (110) planes were observed at 2θ angles of 11.0°, 22.2°, 33.5°, 38.6°, and 59.4°, respectively. In contrast, H‐NiFe‐LDH exhibited XRD peaks assigned to the (003), (006), (112), and (110) planes at 2θ angles of 11.6°, 22.7°, 33.5°, and 59.7°, respectively. Moreover, no XRD peaks corresponding to MIL88A were observed in either sample, indicating the complete transformation of MIL88A to NiFe‐LDH. The (003) peak of H‐NiFe‐LDH (inset of Figure 3a) was observed at a higher 2θ angle than that of S‐NiFe‐LDH, suggesting a decrease in the interlayer distance [32]. This observation indicates that at elevated temperatures, the interlayer anion species change, leading to a decrease in the interlayer distance [34]. In addition, trace Fe_3_O_4_ was formed under high‐temperature conditions (H‐NiFe‐LDH), as displayed in Figure 3a. The reference pattern for NiFe‐LDH (Ni_0.75_Fe_0.25_(CO_3_)_0.125_(OH)_2_⋅0.38H_2_O; JCPDS # 40–0215) and Fe_3_O_4_ (JCPDS # 19–0629) is provided in Figure 3a. Fourier transform infrared spectroscopy (FTIR) was used to predict the molecular functional groups and anionic species in NiFe‐LDH. The FTIR spectra of H‐NiFe‐LDH and S‐NiFe‐LDH revealed several characteristic peaks (Figure 3b). The broad band between 3200 cm^−1^ and 3700 cm^−1^ may be ascribed to the stretching modes of the hydroxyl groups of LDH and interlayer free water molecules. Likewise, the presence of carboxylate anions was evident from the v_s_ (COO─) asymmetric and symmetric stretching around 1600 cm^−1^ and 1400 cm^−1^, respectively [35]. Likewise, another significant peak was observed around 1363 cm^−1^ corresponds to the stretching vibration modes of CO_3_^2−^ ions [36]. These results confirm the presence of carbonate, carboxylate, and water molecules in the interlayers of the S‐NiFe‐LDH and H‐NiFe‐LDH. Additionally, in both materials, a characteristic peak was observed at approximately 593 cm^−1^ which can be attributed to the M‐O stretching vibrations of the metal cations within the LDH structure [37]. Additionally, no residual peaks corresponding to fumaric acid were observed in the H‐NiFe‐LDH and S‐NiFe‐LDH. To validate this, we synthesized a reference carbonate‐intercalated NiFe‐LDH sample via the co‐precipitation method (ESI) at a pH of 10 using NaOH (aq) containing Na_2_CO_3_. The FTIR spectra of H‐NiFe‐LDH and S‐NiFe‐LDH closely match that of the reference NiFe‐LDH, confirming the formation of a carbonate‐type NiFe‐LDH structure rather than an unreacted fumarate or partially decomposed fumaric acid intercalated phase.

The near‐edge X‐ray absorption fine structure (NEXAFS) spectroscopy measurements were conducted to understand the oxidation state and local geometry of H‐NiFe‐LDH and S‐NiFe‐LDH, as shown in Figure 3c,d. The dipole‐allowed electronic transitions from 2p to empty 3d in Ni and Fe could represent the state of the unoccupied d‐orbitals at the transition metal site [38]. Moreover, transition peaks were observed in the low‐energy L_3_ region (2p_3/2_ to 3d) and the higher‐energy L_2_ region (2p_1/2_ to 3d) owing to the spin‐orbit splitting of 2p core‐holes. The L_3_ and L_2_ peaks were further split into two sub‐peaks, corresponding to the electronic transitions from 2p to 3d degenerated t_2g_ and e_g_ orbitals in octahedral coordination under the crystal field effect of the coordinated ligands. The Ni L‐edge spectra illustrated in Figure 3c show sharp L_3_ edge peaks at 852.9 eV with a small shoulder peak at 855.2 eV, which may be assigned to the t_2g_ and e_g_ transitions, respectively, and a broad L_2_ edge peak at ∼871 eV [39, 40, 41]. The Fe L‐edge NEXAFS spectra in Figure 3d revealed L_3_ and L_2_ edge peaks at approximately 709 eV and 722 eV, respectively. The resolved peaks for t_2g_ transitions appeared at 708.2 and 721.1 eV, while e_g_ transitions occurred at 709.6 eV and 722.8 eV. The relative intensities of the e_g_ and t_2g_ peaks in L_3_ serve as indicators of the oxidation state of the material [42]. Moreover, both prepared NiFe‐LDHs with different morphologies showed low e_g_/t_2g_ ratios similar to that of the NiO reference, a signature of the majority Ni^2+^ oxidation states. The S‐NiFe‐LDH displayed a slight increase in peak intensity corresponding to the L_3_ e_g_ transition with a shift toward higher energy, indicating the presence of a slightly higher number of Ni^3+^ compared to H‐NiFe‐LDH [43]. The Fe L‐edge spectra of the NiFe‐LDH materials were similar to those of Fe_3_O_4_, rather than Fe_2_O_3_, indicating the presence of both Fe^3+^ and Fe^2+^ species in the materials [44]. NEXAFS analyses confirm that Fe‐rich NiFe‐LDH with mixed oxidation states of metal ions, including Ni^2+^, Ni^3+^, Fe^2+^, and Fe^3+^, was formed. Further quantification of Ni^2+^, Ni^3+^, Fe^2+^, and Fe^3+^ will be discussed in the XPS analysis.

### 1.2 Electrocatalytic Activities of NiFe‐LDH toward OER

**FIGURE 4:**
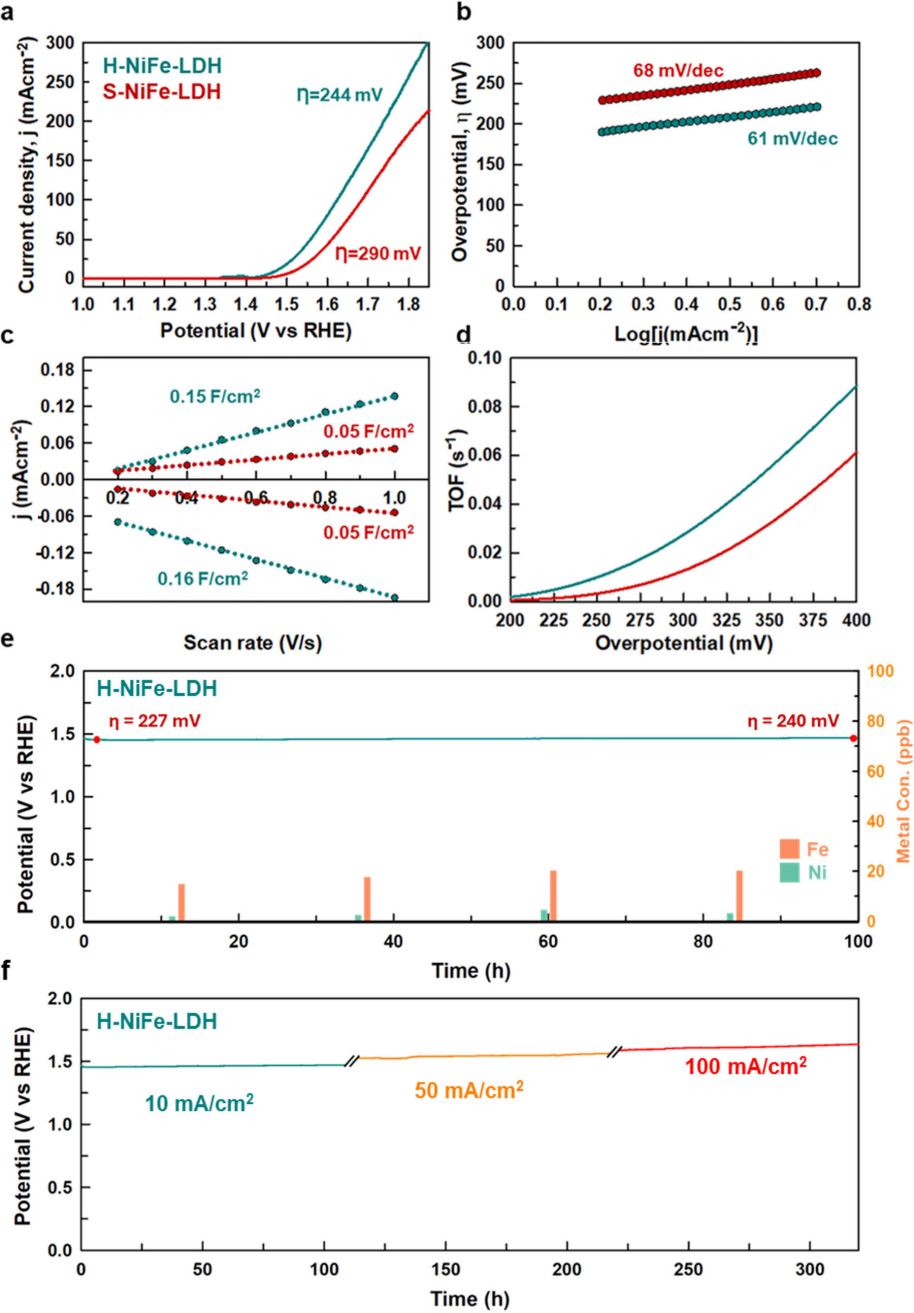
(a) LSV curves, (b) Tafel plots, (c) Electrochemical active surface area, (d) potential‐dependent turnover frequency (TOF) of H‐NiFe‐LDH (dark cyan) and S‐NiFe‐LDH (dark red). (e) Chronopotentiometry stability test of H‐NiFe‐LDH at a constant current density of 10 mA/cm^2^ and ICP‐MS analysis of electrolyte at different time intervals. (f) Chronopotentiometry stability test of H‐NiFe‐LDH at 10 mAcm^−2^,50 mAcm^−2^, 100 mAcm^−2^.

The electrocatalytic OER performance of NiFe‐LDHs with different morphologies was evaluated using a three‐electrode configuration, with a Hg/HgO reference electrode, a Pt‐plate as the counter electrode, and the catalyst (300 µgcm^−2^) loaded onto carbon‐fibre paper as the working electrode, in 1 m KOH (aq) electrolyte. The iR‐compensated (95%) linear sweep voltammetry (LSV) measurements were performed at a scan rate of 1 mV/s for H‐NiFe‐LDH and S‐NiFe‐LDH samples. The LSV curve (Figure 4a) for H‐NiFe‐LDH displayed a lower onset potential and required an overpotential (η) of 244 mV (vs. RHE) at a current density of 10 mA/cm^2^. Notably, this performance surpasses that of S‐NiFe‐LDH, which requires a higher overpotential of 290 mV. The H‐NiFe‐LDH exhibited a superior current density at higher potentials (Figure S4), which can be attributed to its hollow morphology and increased availability of active sites. In addition, the OER kinetics were examined using the Tafel slope plot derived from the LSV curves (Figure 4b) to understand how rapidly the reaction rate increased with the overpotential. H‐NiFe‐LDH showed enhanced reaction kinetics, including a faster kinetic rate and higher electron transfer efficiency, as evidenced by its lower Tafel slope of 61 mVdec^−1^, compared to S‐NiFe‐LDH (68 mVdec^−1^). Furthermore, the electrochemically active surface area (ECSA) of the samples was determined using double‐layer capacitance, as shown in Figure 4c. The study, based on the CV measurements at different scan rates (Figure S5) in the non‐faradaic region ranging from 1.065 V to 1.165 V (vs RHE), exhibited a double‐layer capacitance of 0.15 mF/cm^2^ in H‐NiFe‐LDH, which is threefold higher than that of S‐NiFe‐LDH (0.05 mF/cm^2^). H‐NiFe‐LDH displayed a pronounced reduction in the charge transfer resistance, as revealed by electrochemical impedance spectroscopy (Figure S6a), indicating that H‐NiFe‐LDH has a higher conductivity than S‐NiFe‐LDH. This is attributed to the formation of a hollow nanocapsule morphology, which creates a more effective electron transport pathway. Furthermore, to investigate the intrinsic nature of the electrocatalyst, the turnover frequency (TOF, per site) was evaluated from the LSV curve based on the number of active metal sites determined using ICP‐OES measurements. As shown in Figure 4d, the TOF of H‐NiFe‐LDH is higher than that of S‐NiFe‐LDH, where we assume that all the metal atoms behave as active sites for electrocatalysis.

The long‐term stability of the electrocatalysts was evaluated using chronopotentiometry measurements. The working electrode was prepared by coating a catalyst slurry onto a 1 cm^2^ area of a nickel foam support. Figure 4e shows the chronopotentiometry curve of the H‐NiFe‐LDH measured at a current density of 10 mAcm^−2^. The electrocatalyst exhibited an overpotential of 227 mV at 1 h, which increased to 240 mV after an extended 100 h chronopotentiometry test, demonstrating the excellent durability of the developed electrocatalyst. Generally, when the catalyst undergoes the LOM pathway, a large amount of LO is consumed, and metal ion leaching into the electrolyte solution occurs, thereby reducing OER stability. In contrast, the high stability of H‐NiFe‐LDH indicates balanced LO consumption and regeneration, as well as minimal leaching of Fe ions from the electrocatalyst, which is a major challenge for similar catalysts. The rates of Fe and Ni leaching were determined using periodic ICP‐MS analysis of the electrolyte samples collected during the stability test (Figure 4e). After 100 h, the Fe and Ni concentrations in the electrolyte were ∼ 25 ppb and 2 ppb, respectively, indicating the excellent stability of the NiFe‐LDH catalyst, which is evident from the minimal increase in overpotential. Moreover, after the initial slight increase in Fe and Ni leaching (∼ 12 h), further changes were negligible. Additionally, even at a higher current density of 50 mAcm^−2^, the catalyst showed a minimal overpotential increase of only 34 mV after the 100 h stability test (Figure 4f). During stability test at 50 mAcm^−2^ and 100 mAcm^−2^, the observed increase in overpotential was primarily attributed to the partial peeling/delamination of the catalyst from the electrode surface by vigorous oxygen bubble evolution, rather than the degradation of the catalyst material. The electrolyte was replaced between each stability test. After the stability test (10 mAcm^−2^, 10 h), the catalysts were further investigated via XRD, FTIR, and SEM analyses, which are displayed in Figure S7. XRD patterns of the catalysts before and after electrocatalysis showed no significant difference, indicating that the crystal structure was maintained and that the materials were electrochemically stable. FTIR spectra showed the presence of OH groups and vibrations of intercalated water, which can be confirmed by the slightly lower‐angle shift on the (003) peak of NiFe‐LDH in XRD patterns. Furthermore, the SEM images revealed no significant degradation in morphology and size, indicating that the samples (Figure S7c,d) maintained their crystal structure and morphology over prolonged OER measurements. The high TOF, low Tafel slope, reduced charge transfer resistance and excellent stability of the H‐NiFe‐LDH electrocatalyst imply its superiority in electrocatalytic oxygen generation. Table S4 provides a comparative analysis of the OER activities observed in the current study and those of recently reported electrocatalysts. As shown in Table S4, the Fe‐rich NiFe‐LDH displayed the highest OER activity among similar reported materials, demonstrating the role of the structure and the importance of active sites in enhancing the electrocatalytic activity.

**FIGURE 5:**
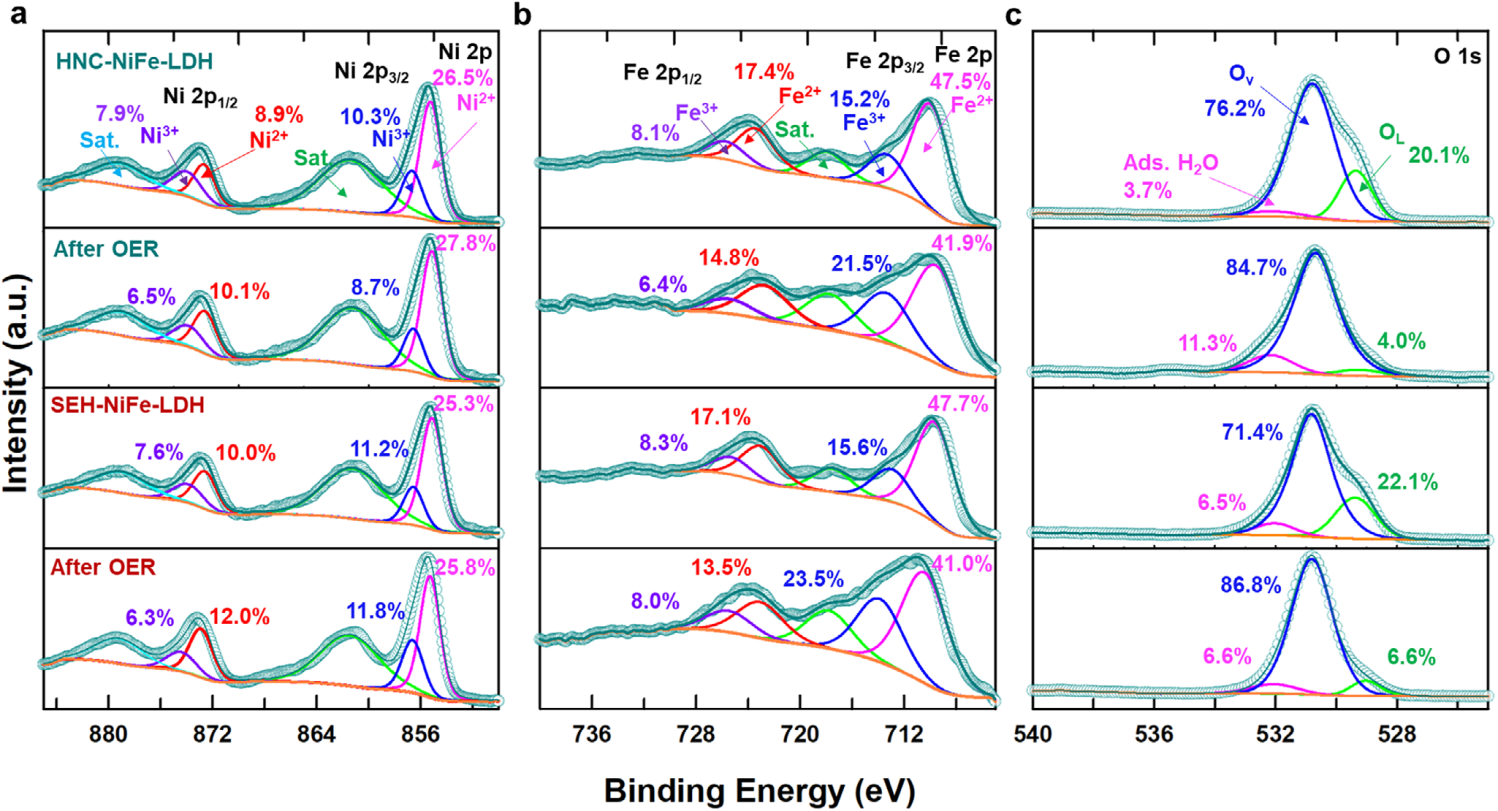
XPS spectra of H‐NiFe‐LDH and S‐NiFe‐LDH before and after the OER analysis: (a) Ni 2p, (b) Fe 2p, and (c) O 1s spectra.

High‐resolution XPS analysis was conducted to investigate changes in the chemical composition and elemental valences of NiFe‐LDH before and after OER and to explore the electrocatalytic reaction pathway of the OER catalyst. The XPS survey spectrum revealed the presence of Ni, Fe, O, and C (Figure S8). The high‐resolution Ni 2p spectra of the NiFe‐LDH samples exhibited two distinct peaks corresponding to Ni 2p_1/2_ and Ni 2p_3/2,_ which were deconvoluted into four peaks located at 872.7 eV, 874.1 eV, 855.2 eV, and 856.6 eV for H‐NiFe‐LDH and S‐NiFe‐LDH, as illustrated in Figure 5a (Table S1). The peaks at 879.1 eV and 861.3 eV can be assigned to the satellite peaks corresponding to the Ni 2p_1/2_ and Ni 2p_3/2_ transitions arising from electron‐electron interactions and shake‐up processes. The peaks with binding energies of 872.7 eV and 855.2 eV can be ascribed to the Ni^2+^ species, while the peaks at higher binding energies of 874.1 eV and 856.6 eV can be attributed to the Ni^3+^ species [45]. In the high‐resolution XPS spectra of Fe 2p, two distinct peaks (Fe 2p_3/2_ and Fe 2p_1/2_) are observed, as shown in Figure 5b. The Fe 2p_3/2_ is not only narrower but also more intense than that of Fe 2p_1/2_. The Fe 2p_3/2_ peak has an associated shake‐up satellite peak at 718.2 eV which is distinguishable and located approximately 7.7 eV higher than the main Fe 2p_3/2_ peak. The peak area of Fe 2p_3/2_ was larger than that of Fe 2p_1/2,_ and the corresponding peaks were deconvoluted to binding energies of 723.4 eV and 725.8 eV and 710.6 eV and 713.9 eV (Figure 5b), representing the contributions of Fe^2+^ and Fe^3+^ species in the LDH structure (Table S2) [19, 46]. In summary, the presence of Fe^2+^, Fe^3+^, Ni^2+,^ and Ni^3+^ in H‐NiFe‐LDH and S‐NiFe‐LDH was verified via XPS analysis, which is in agreement with the NEXAFS analysis. The high‐resolution XPS O 1s spectra of NiFe‐LDH were deconvoluted into three peaks at 529.3 eV, 530.7 eV, and 532.1 eV, which were assigned to lattice oxygen (O_L_), oxygen vacancy defects (O_V_), and surface‐adsorbed H_2_O molecules, respectively, as shown in Figure 5c, and the details are summarized in Table S3. [47] The generation of abundant O_V_ is due to the distortions caused by abundant Fe atoms on the Ni‐LDH framework. The high O_V_ defects in H‐NiFe‐LDH were further confirmed by electron paramagnetic resonance (EPR) measurements in comparison to the reference NiFe‐LDH (Figure S9) [47]. Oxygen vacancy defects can facilitate OH^−^ absorption and influence the adsorption free energy of OER intermediates, thereby boosting overall OER kinetics. The concentration of O_V_ defects in H‐NiFe‐LDH was significantly higher than that in S‐NiFe‐LDH, providing additional support for the observed lower overpotential in H‐NiFe‐LDH [48]. As evaluated from the deconvoluted Ni 2p spectra, the Ni^3+^/Ni^2+^ ratios of H‐NiFe‐LDH and S‐NiFe‐LDH were found to be 0.51 and 0.53, respectively, before the OER measurements, while these ratios were 0.40 and 0.47 for both after the OER measurement, indicating the reduction of nickel from 3+ to 2+ during the OER. In the case of Fe 2p, the Fe^3+^/Fe^2+^ ratios of H‐NiFe‐LDH and S‐NiFe‐LDH were determined to be 0.36 and 0.37, respectively, before the OER measurements, while their Fe^3+^/Fe^2+^ ratios significantly increased to 0.49 and 0.57, respectively, after the OER, suggesting the partial oxidation of Fe^2+^ to Fe^3+^ during the OER. Equations (1) and (2) below corroborate ICP‐OES and ex‐situ analysis to illustrate the change in oxidation state after the OER analysis.

(1) $$\begin{array}{ccc} & & H-\mathrm{NiFe}-\mathrm{LDH}-{\mathrm{Ni}}_{0.61}{\mathrm{Fe}}_{0.39}\\ & & {\left[{\mathrm{Ni}}^{2+}\right]}_{0.40}\;{\left[{\mathrm{Ni}}^{3+}\right]}_{0.21}{\left[{\mathrm{Fe}}^{2+}\right]}_{0.29}\;{\left[{\mathrm{Fe}}^{3+}\right]}_{0.10}\;\\ & & \;\to {\left[{\mathrm{Ni}}^{2+}\right]}_{0.43}\;{\left[{\mathrm{Ni}}^{3+}\right]}_{0.18}{\left[{\mathrm{Fe}}^{2+}\right]}_{0.26}\;{\left[{\mathrm{Fe}}^{3+}\right]}_{0.13} \end{array}$$

(2) $$\begin{array}{ccc} & & S-\mathrm{NiFe}-\mathrm{LDH}-{\mathrm{Ni}}_{0.68}{\mathrm{Fe}}_{0.32}\\ & & {\left[{\mathrm{Ni}}^{2+}\right]}_{0.44}\;{\left[{\mathrm{Ni}}^{3+}\right]}_{0.24}{\left[{\mathrm{Fe}}^{2+}\right]}_{0.23}\;{\left[{\mathrm{Fe}}^{3+}\right]}_{0.09}\;\;\\ & & \;\to \;{\left[{\mathrm{Ni}}^{2+}\right]}_{0.46}\;{\left[{\mathrm{Ni}}^{3+}\right]}_{0.22}{\left[{\mathrm{Fe}}^{2+}\right]}_{0.20}\;{\left[{\mathrm{Fe}}^{3+}\right]}_{0.12}\; \end{array}$$

In both cases, the Ni^2+^ and Fe^3+^ populations slightly increased, whereas the Ni^3+^ and Fe^2+^ populations decreased. Since the Fe^2+^ is oxidised to form Fe^3+^ during the OER, the Fe sites act as AEM sites in S‐NiFe‐LDH and H‐NiFe‐LDH, which is consistent with previous reports [19]. Notably, higher oxidation states of Fe and Ni species were not observed, which may be attributed to the inherent limitations of ex‐situ experiments, as Fe^4+^ and above are highly unstable [49]. Based on the XPS results, it should be noted that the incorporation of abundant Fe in NiFe‐LDH could decrease the oxidation state of Ni, which makes it more prone to water oxidation and further promotes OH^−^ adsorption, thereby enhancing the OER efficiency. In addition, a large number of O_V_ defects were observed in the O 1s spectra of the NiFe‐LDH samples, S‐NiFe‐LDH and H‐NiFe‐LDH. Furthermore, after the OER, the number of O_V_ defects increased, and the intensity of the peak corresponding to the O_L_ decreased, indicating the breaking of the metal‐oxygen bond and the release of O_L_, which is a signature process in LOM for OER catalysis [21]. The lattice oxygen sites actively participate in the reaction, creating transient O_V_ defects which are subsequently replenished back to O_L_, allowing the catalyst surface to reach a steady‐state equilibrium during the reaction. For H‐NiFe‐LDH and S‐NiFe‐LDH, the O_V_ at this equilibrium was higher than that of the as‐synthesised electrocatalyst. In conclusion, comparative XPS analysis before and after OER indicates that H‐NiFe‐LDH and S‐NiFe‐LDH predominantly follow a LOM pathway, driven by abundant oxygen vacancies and changes in lattice oxygen species, whereas at a higher overpotential, an Fe^3+^−mediated AEM reaction pathway may also contribute.

**FIGURE 6:**
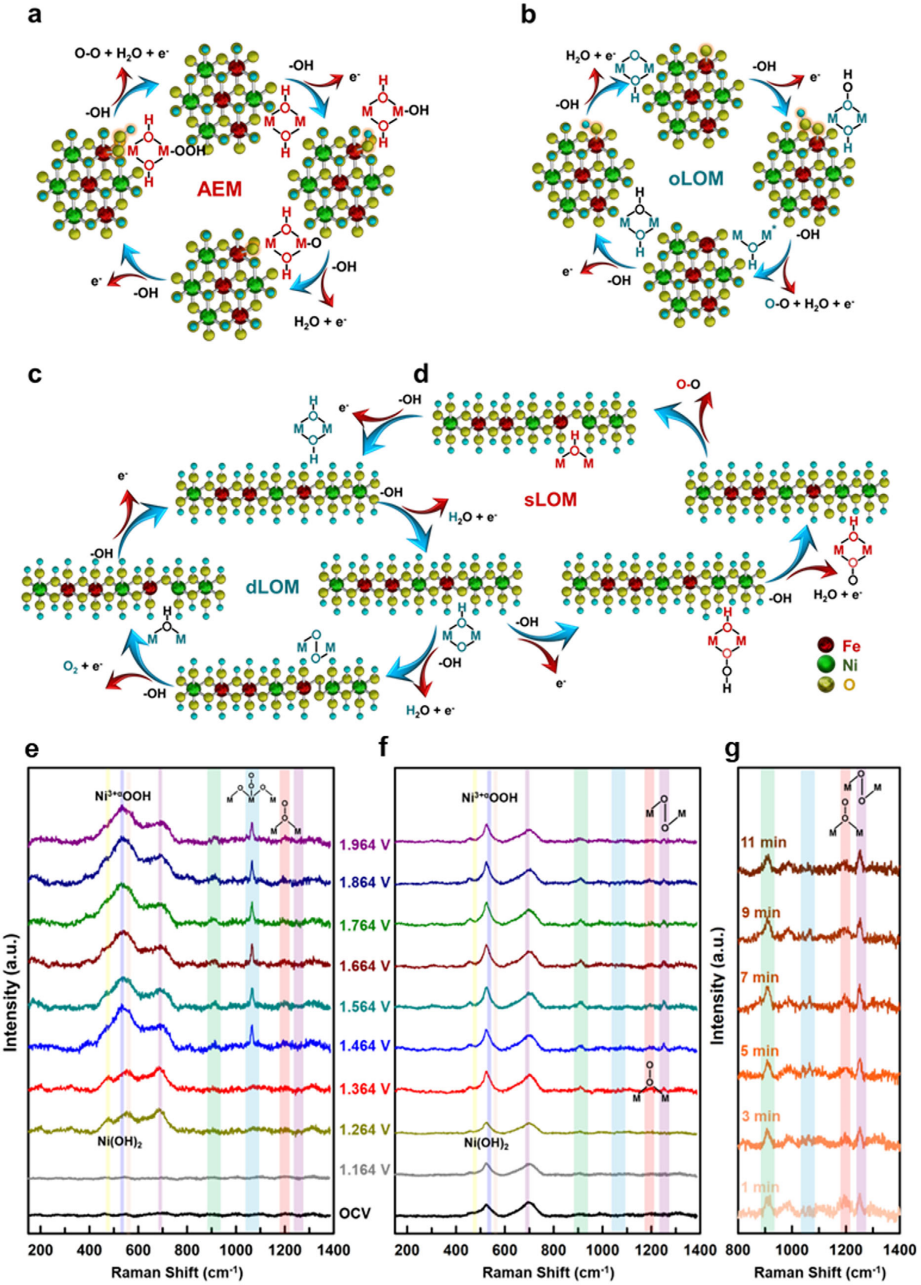
Schematic illustration of possible OER pathways for Fe‐rich NiFe‐LDH: (a) AEM, (b) oLOM, (c) dLOM, and (d) sLOM. In‐situ Raman spectra of (e) S‐NiFe‐LDH, (f) H‐NiFe‐LDH at different applied potential (g) In‐situ Raman spectra of H‐NiFe‐LDH recorded at a constant potential of 1.464 V at different time intervals.

The possible electrocatalytic OER pathways of absorbate evolution mechanism (AEM), oxygen vacancy site LOM (oLOM), single metal site LOM (sLOM), and dual metal site LOM (dLOM) are illustrated in Figure 6a–d [18, 20, 50]. The AEM is a widely acknowledged mechanism involving adsorption‐deprotonation‐coupling‐desorption of oxygen intermediate species on metal active sites, as displayed in Figure 6a [51]. The high binding energy of oxygen intermediates on active metal sites imposes the theoretical overpotential limit of 340 mV for AEM, because the strong binding can lead to a slow desorption step, which suppresses the overall rate of OER. However, NiFe‐LDH catalysts exhibited overpotentials lower than 340 mV, indicating that there may be other factors contributing to the high activity of NiFe‐LDH in delivering enhanced OER performance [21]. The abundant oxygen vacancies, high oxidation state of metal elements, low overpotential, and significant alterations in the metal‐to‐oxygen coordination environment after OER catalysis collectively suggest the predominant involvement of the LOM in redox chemistry. In contrast to the AEM pathway, where metal active sites remain stable while the oxidation states change actively, the LOM pathway shows a dynamic change in the active sites during the OER process. The LO sites undergo repeated oxidation, exchange, and release during catalysis. The generated oxygen vacancy sites bind with OH^−^ from the electrolyte to form new LO sites. Subsequent LO activation is necessary to repeat this process, which is a prerequisite for any LOM pathway. In the oLOM pathway (Figure 6b), LO is activated, increasing electrophilicity and facilitating subsequent nucleophilic attack from OH‐ in the electrolyte to form M‐OOH. Furthermore, the release of gaseous O_2_ species generates more oxygen vacancies, which are replenished with OH‐ species to regenerate the LO sites. The sLOM and dLOM mechanisms involve LO attached to single and dual metal sites on the catalyst surface (Figure 6c,d). The sLOM undergoes direct coupling of activated LO with an intermediate oxygen species to generate M‐OO^*^, which subsequently generates gaseous O_2_. However, in dLOM, the neighbouring active LO undergoes intermolecular coupling to form M‐OO‐M species, and subsequent oxidation generates gaseous oxygen, as schematically represented in Figure 6c. The LOM mechanism is more efficient than the AEM because it does not require the formation of additional surface‐bound ^*^OOH intermediates, which are known to be unstable [19, 21, 50, 51].

To further understand the OER reaction mechanism on the NiFe LDH catalyst, in‐situ Raman analysis was performed (Figure 6e,f). We used a 0.1m KOH electrolyte, Ag/AgCl reference electrode, and NiFe‐LDH‐loaded carbon fibre paper as the working electrode. Raman signals were recorded with a change in potential to understand the change in surface activity. With the increase in potential, the S‐NiFe‐LDH underwent dynamic surface reconstruction, and several new peaks emerged in the range of 400–800 cm^−1^, corresponding to the M─O bonds (M ═ Ni/Fe). During electrocatalysis, some Ni^α+^/Fe^α+^ leaches out to form Ni_2_FeO_4_, whose characteristic A_1g_ stretching mode can be observed at 695 cm^−1^ [52]. Furthermore, at 1.464 V, a sharp peak emerges at 1067 cm^−1^, indicating the formation of a Ni‐OO^−^ group even at lower overpotentials, which suggests fast kinetics in the AEM or an oLOM pathway [53]. To elucidate the underlying mechanism, theoretical calculations were performed and are discussed in the next section. The identified OER intermediates provide insights into possible reaction pathways. The formation of negatively charged peroxo‐like species (O_2_^2−^ and O_2_^−^) provides indirect evidence for the LOM mechanism. Xie et al. conducted computational studies on NiFe oxides and identified two stable peroxo‐like species, bridge‐mode and top‐mode, with calculated Raman bands at 1076 cm^−1^ and 1237 cm^−1^, respectively. However, in the in‐situ Raman measurements, these features appear at much higher wavenumber [54]. These signatures enable differentiation between the sLOM (top‐mode) and dLOM (bridge‐mode) pathways. In S‐NiFe‐LDH (Figure 6e), a low‐intensity broad peak that emerged at 1206 cm^−1^ can be reasonably assigned to the top O‐O^−^ intermediate species, indicating the prominent sLOM mechanism. No significant peak corresponding to the M‐O‐O‐M intermediate (bridge O‐O^−^) was observed, suggesting the absence of the dLOM pathway. In contrast, H‐NiFe‐LDH exhibits noticeable changes in Raman peak intensity between 400 cm^−1^ and 800 cm^−1^ at higher overpotentials, corresponding to the M‐O vibrational modes (Figure 6f). Moreover, at a higher overpotential (V = 1.464 V), a new pronounced peak appears at 1254 cm^−1^, which can be assigned to the M‐O‐O‐M intermediate (bridge O‐O^−^), indicating a prominent dLOM pathway. A relatively weaker peak at ∼1202 cm^−1^ was also observed, corresponding to the presence of the top O‐O^−^ intermediate species, suggesting a minor contribution from the sLOM mechanism. This was further confirmed by in‐situ Raman measurements conducted at a constant potential of 1.464 V for an extended duration (Figure 6g), which improves spectral resolution. Interestingly, the peak at 1067 cm^−1^, which was prominent in S‐NiFe‐LDH, was completely absent in H‐NiFe‐LDH, even under prolonged applied bias, highlighting the distinct reaction pathways of the two catalysts.

### 1.3 Theoretical Insights on NiFe‐LDH

#### 1.3.1 Crystal Structures and Electronic Properties of Ni_1‐x_Fe_x_‐LDH (x = 12.5%, 25%)

**FIGURE 7:**
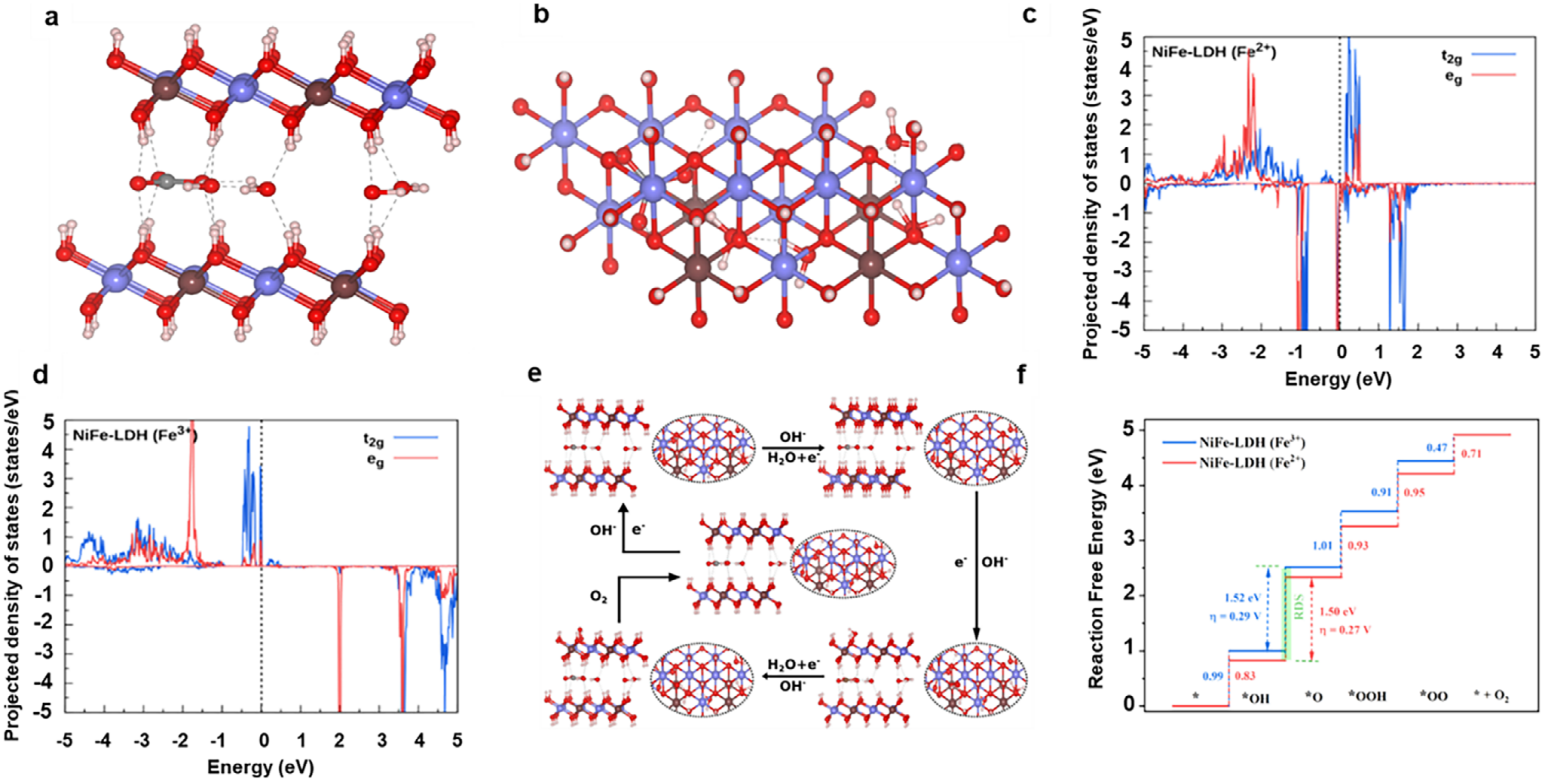
(a) Side and (b) top views of the optimized configuration of Ni_6_Fe_2_‐LDH. Brown, purple, red, grey, and light pink denote Fe, Ni, O, C, and H atoms, respectively. Projected Density of States of Fe‐d orbital for Ni_6_Fe_2_‐LDH (c) Fe^2+^ and (d) Fe^3+^. The red and blue lines represent the e_g_ and t_2g_ orbitals, respectively, and the Fermi level is shifted to zero. (e) Proposed 4e^−^ mechanism of LOM (Ni^3+^−O−Fe^2+^) reaction along with the optimized structures of Ni_6_Fe_2_‐LDH. (f) The Gibbs free energy diagrams of sLOM pathways on NiFe‐LDH, where the arrows show the rate‐determining steps (RDS).

We performed theoretical calculations based on density functional theory (DFT), which focused on the modelling of a NiFe‐LDH configuration. Our investigation aims to calibrate the Fe content in the NiFe‐LDH, which is a crucial factor for observing the occurrence of mixed oxidation states (OS) in the proposed structure. We modeled the structure with a Fe concentration of 12.5%, in which one Ni atom was replaced by one Fe atom, and with a 25% concentration, two Ni atoms were replaced with two Fe atoms in Ni_1‐x_Fe_x_‐LDH. H_2_O and ${\mathrm{CO}}_{3}^{2-}$ ions were intercalated between the two layers and further connected with the Ni_6_Fe_2_‐LDH layers (see Figure 7a,b; Figure S10a,b). The calculated lattice constant and interlayer distances were 3.10 Å and 7.7 Å for NiFe‐LDH, respectively, which is consistent with an earlier study [17]. The total Density of States (TDOS) for the Ni_7_Fe_1_‐LDH and Ni_6_Fe_2_‐LDH configurations is shown in Figure S11. To gain more insight, we calculated the projected density of states (PDOS) (Figure 7c,d; Figure S10c,d) of the Ni_6_Fe_2_‐LDH and Ni_7_Fe_1_‐LDH structures. As shown in Figure S10c,d, the Fe atom is in the 3+ OS for Ni_7_Fe_1_‐LDH at a 12.5% Fe concentration. However, the Fe atoms in Ni_6_Fe_2_‐LDH (Fe at 25% concentration) (Figure 7c,d) were found to be in a mixed 2+ and 3+ OS. The oxidation states of Ni atoms near Fe in Ni_6_Fe_2_‐LDH and Ni_7_Fe_1_‐LDH were found to be 3+. The Fe atom on the edge of a bilayer was found to be in the 2+ OS, whereas the central Fe atom was found to be in the 3+ OS. As shown in Figure 7c, the presence of t_2g_ and e_g_ spin‐down states near the Fermi level indicates that one of the Fe atoms is in the 2+ OS. However, we observed that the t_2g_ states were absent near the Fermi level of the second Fe atom, as shown in Figure 7d. This validates the existence of mixed OS (Fe^3+^/Fe^2+^) in the Ni_6_Fe_2_‐LDH, which is consistent with the previous study [55].

#### 1.3.2 The Catalytic Oxygen Evolution Reaction Mechanism

DFT calculations were conducted to gain insights into the enhanced OER through the sLOM pathway for the mixed oxidation states of Fe atoms (2+ and 3+) in Ni_6_Fe_2_‐LDH. In an alkaline environment, the typical OER process can be expressed by the following elementary reaction paths:

(3)

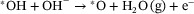



(4)

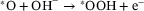



(5)

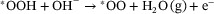



(6)

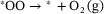



(7)

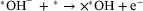




Here, (g) denotes the gas phase, and * denotes the catalyst surface, while ^*^OH, ^*^O, ^*^OOH and ^*^OO represent adsorbed intermediates. Figure 7e depicts the adsorption configurations of the intermediate species and the detailed LOM, highlighting the multiple steps involved. To evaluate the OER activities of the Ni_6_Fe_2_‐LDH catalysts, we investigated the free energies of the OER intermediates, as depicted in Figure 7f. In the LOM pathway, the thermodynamic overpotential on the LO site (Ni^3+^−O−Fe^3+^) atom for the Ni_6_Fe_2_‐LDH system was 0.29 V, whereas the calculated thermodynamic overpotential on the LO site (Ni^3+^−O−Fe^2+^) was 0.27 V. This indicates higher LO activity at (Ni^3+^−O−Fe^2+^) than at (Ni^3+^−O−Fe^3+^) in the Ni_6_Fe_2_‐LDH system [56]. The deprotonation of ^*^OH to ^*^O is the rate‐determining step (RDS) for Ni^3+^−O−Fe^3+^, with a ΔG_2_ value of 1.52 eV. In contrast, for Ni^3+^−O−Fe^2+^, the free energy change for the first step was 1.50 eV. Our computed results suggest that the overpotential of the LO atom near Fe^3+^ is slightly higher than that of the LO atom close to Fe^2+^ owing to the strong interaction between the LO, Fe/Ni atoms, and intermediate species, which results in a higher energy barrier. These results are consistent with the experimental OER activity data, emphasizing the significant contribution of mixed Fe^2+^ and Fe^3+^ OS in NiFe‐LDH to the enhancement of catalytic performance. To provide more support to our results, we have further calculated the d‐band center and Bader charge for the Ni^3+^−O−Fe^2+^ and Ni^3+^−O−Fe^3+^ configurations. The Bader charge did not show a significant change in the charge transfer of ^*^O and ^*^OH species for Ni^3+^−O−Fe^2+^ and Ni^3+^−O−Fe^3+^. However, the d‐band center analysis shows that Ni^3+^−O−Fe^2+^ has a value of −2.240 eV, while Ni^3+^−O−Fe^3+^ shifts closer to the Fermi level at −2.074 eV, indicating stronger ^*^O binding at Fe^3+^ sites. This stronger binding correlates with a slightly higher energy barrier for Fe^3+^ sites, reinforcing that Fe^2+^ sites facilitate OER more favourably. Although operando validation is challenging, these computational insights supported by electronic structure analysis, bond length variations, and d‐band shifts provide a mechanistic rationale for Fe^2+^−induced modulation of Ni─O─Fe bonds and their role in activating lattice oxygen sites.

In summary, we successfully developed Fe‐rich NiFe‐LDHs with unique morphologies derived from Fe‐based metal‐organic frameworks to introduce LO vacancies and modulate the O 2p Fermi levels to activate the OER LOM pathway. The high Fe content of the NiFe‐LDH catalyst promoted the stabilization of Fe^2+^ alongside Fe^3+^, resulting in remarkable OER performance, including a lower overpotential of 244 mV at a current density of 10 mA/cm^2^, a small Tafel slope of 61 mVdec^−1^ indicative of fast charge transfer kinetics, and high catalytic stability, outperforming many of the best reported catalysts. Ex situ XPS analysis of the developed NiFe‐LDH catalysts before and after the OER reveals the active involvement of lattice oxygen and oxygen vacancy sites as active sites for OER catalysis. The observed changes in the Fe oxidation states further suggest the contribution of Fe‐centered active sites via an AEM pathway at elevated overpotentials. In situ Raman spectroscopy revealed negatively charged peroxo‐like species (O_2_^2−^ and O_2_^−^) at 1254 cm^−1^ and 1202 cm^−1,^ which pinpointed the s‐LOM and d‐LOM pathways in Fe‐rich NiFe‐LDH. Computational analysis corroborates the stabilization of Fe^2+^ at high Fe loadings (>25%) and reveals enhanced lattice oxygen activity at Ni^3+^−O−Fe^2+^ sites compared to that of Ni^3+^−O−Fe^3+^.

## Conflicts of Interest

The authors declare no conflict of interest.

## Supporting information




**Supporting File**: advs73680‐sup‐0001‐SuppMat.docx.

## Data Availability

The data that support the findings of this study are available from the corresponding author upon reasonable request.
