# Supplementary material for: Fe2+‐ Induced Activation of Single and Dual Metal Site—Lattice Oxygen Mechanism in Fe Rich NiFe‐LDHs for Oxygen Evolution Reaction
Source: Adv Sci (Weinh). 2026 Mar 4;13(30):e21259. doi: 10.1002/advs.202521259 (PMC13248766; doi:10.1002/advs.202521259)
Supplement: Supplementary file 1 — Supporting File: advs73680‐sup‐0001‐SuppMat.docx. [file ADVS-13-e21259-s001.docx]

**Electronic supplementary information (ESI)**

**Fe^2+^- Induced activation of Single and Dual Metal Site - Lattice Oxygen Mechanism in Fe rich NiFe-LDHs for Oxygen Evolution Reaction**

Nithinraj Panangattu Dharmarajan, Mohammed Fawaz, Vanshree Parey, Sudip Chakraborty, CI Sathish, Thi Thuy Kieu Tran, Xuan Minh Chau Ta, Siddulu Naidu Talapaneni, Zhixuan Li, Kavitha Ramadass, Antonio Tricoli, Jae-Hun Yang*, Ajayan Vinu*

Nithinraj Panangattu Dharmarajan, Mohammed Fawaz, CI Sathish, Siddulu Naidu Talapaneni, Zhixuan Li, Kavitha Ramadass, Jae-Hun Yang, Ajayan Vinu

*Global Innovative Centre of Advanced Nanomaterials, College of Engineering, Science and Environment, University of Newcastle, Callaghan, 2308, NSW, Australia.*

Email: [ajayan.vinu@newcastle.edu.au](mailto:ajayan.vinu@newcastle.edu.au)

Thi Thuy Kieu Tran, Xuan Minh Chau Ta, Antonio Tricoli

*Nanotechnology Research Laboratory Faculty of Engineering University of Sydney, NSW 2006, Australia*

Vanshree Parey, *Sudip Chakraborty*

*Materials Theory for Energy Scavenging (MATES) Lab, Harish-Chandra Research Institute (HRI), Allahabad, HBNI, Chhatnag Road, Jhunsi, Prayagraj (Allahabad) 211019 India*

**Materials and Chemicals:** Iron (III) nitrate nonahydrate (Fe(NO_3_)_3_·9H_2_O), Nickel(II) nitrate hexahydrate (Ni(NO_3_)_2_·6H_2_O), Fumaric acid, Hexamethylenetetramine (HMTA), Carbon fibre paper, Nafion (5 wt%), Na_2_CO_3_, NaOH, KOH were purchased from Merck Sigma-Aldrich and used without further purification.

**Synthesis of MIL88A:** 3 mmol of iron (III) nitrate nonahydrate (Fe(NO_3_)_3_·9H_2_O) and 3 mmol of fumaric acid were dissolved in 140 ml of water. The prepared solution was transferred to a Teflon-lined stainless-steel autoclave and kept in a preheated oven at 110 ^o^C for 6 h. The final product was centrifuged, washed with ethanol and water, and dried at 100 ^o^C for 12 h in a convection oven.

**Synthesis of Hollow Nano Capsule-like NiFe-LDH (H-NiFe-LDH):** 1 mmol of nickel nitrate hexahydrate and 1 mmol of HMTA were dissolved in 140 ml of water. 300 mg of MIL88A was dispersed in the above reaction medium, transferred to a Teflon-lined stainless-steel autoclave, and kept in a preheated oven at 180 ^o^C for 12 h. The final product was centrifuged, washed in ethanol and water and dried at 100 ^o^C for 12 h in a convection oven.

**Synthesis of sheet-encapsulated hollow NiFe-Layered Double Hydroxide (S-NiFe-Layered Double Hydroxide):** 3 mmol of nickel nitrate hexahydrate and 3 mmol of hexamethylenetetramine (HMTA) were dissolved in 140 ml of water. 300 mg MIL88A was dispersed in the above reaction medium, transferred to a Teflon-lined stainless-steel autoclave, and kept in a preheated oven at 120 ^o^C for 12 h. The final product was centrifuged, washed in ethanol and water and dried at 100 ^o^C for 12 h in a convection oven.

**Synthesis of Nickel hydroxide nanosheets (NiOH nano sheets):** 3 mmol of nickel nitrate hexahydrate and 3 mmol of HMTA were dissolved in 140 ml of water. The reaction medium was transferred to a Teflon-lined stainless-steel autoclave and kept in a preheated oven at 120 ^o^C for 12 h. The final product was centrifuged, washed in ethanol and water and dried at 100 ^o^C for 12 h in a convection oven.

**Synthesis of carbonate-intercalated NiFe-LDH:**

The reference carbonate-intercalated NiFe-LDH was prepared by co-precipitating Ni(NO_3_)_2_.6H_2_O and Fe(NO_3_)_3_.9H_2_O (4:1 molar ratio) by adjusting pH 10.0 (± 0.5) using 0.5M with NaOH containing in Na_2_CO_3_. The resulting suspension was stirred at room temperature and aged for 12h. The precipitate was washed repeatedly with deionized water to remove the excess salts and unreacted metal ions, followed by freeze-drying to obtain fine NiFe-LDH powder.

**Material characterization:**

The morphological and textural properties were characterized using a JEOL JSM-7900F Field-Emission scanning electron microscope (FESEM) and a JEOL JEM-F200 transmission electron microscope (TEM). A Panalytical Empyrean X-ray diffractometer was used to obtain powder X-ray diffraction patterns to analyze the crystal structure of the sample (Cu-Kα radiation (λ = 1.5418 Å), Galipix Detector, and operated at 40 kV and 40 mA). Nicolet^TM^ iS^TM^ 10 FTIR spectrometer equipped with Attenuated Total Reflectance (ATR) was used to determine the chemical bonding and interlayer anions in the LDH structure. N_2_ adsorption-desorption isotherm analysis was performed using a Micromeritics ASAP 2040 analyzer to study the surface area and pore size of the catalyst. Near-Edge X-ray Absorption fine structure (NEXAFS) studies were carried out at the Soft X-ray Spectroscopy Beamline at the Singapore Synchrotron Light Source to analyze the local coordination of the catalyst. X-ray photoelectron spectroscopy (XPS) measurements were conducted using monochromatized Al Kα radiation to understand the surface elemental composition, chemical bonding, coordination environment, and oxidation state of the catalyst.

**Fabrication of anode:** 5 mg of catalyst and 50 µl of 5 wt% Nafion were dispersed in 950 µl of isopropanol. The prepared suspension was sonicated for 5 min to obtain a uniform slurry. The above slurry (60 µL) was drop-cast onto a 1 × 1 cm^2^ area of a pre-cleaned carbon fiber paper strip (AvCarb® MGL190). The electrode was naturally dried overnight under ambient conditions.

**Evaluation of Electrocatalytic OER:** A CH instrument (CHI770D) electrochemical workstation was used to test the OER performance of the material. All measurements were carried out in a 3-electrode configuration where a platinum spring electrode was used as the counter electrode, Hg/HgO was used as the reference electrode and 1M KOH aqueous solution was used as the electrolyte. The formula given below is used to convert the measured potential to RHE.

V_RHE_ = V_Hg/HgO_ + 0.098 V + pH × (0.059) - (1)

The electrolyte was purged and saturated with Ar gas for 15 min before the experiments. Before any measurement, the electrodes were stabilized by scanning 50 cycles of CV at a scan rate of 50 mV/s. LSV curves were collected at 1 mVs^-1^ and compensated for IR drop (95%). The overpotential (η) was calculated using

η (V) = E (RHE) – 1.23 V. - (2)

Electrochemical impedance spectroscopy (EIS) was performed from 0.1 to 10^5^ Hz with 1.465 V (vs RHE). The stability of the electrocatalyst was evaluated using chronopotentiometry measurements. In chronopotentiometry measurements, a fixed current density, typically 10mA cm^-2^, was applied to the working electrode to assess the current generated from the faradic process.

**Electrochemical active surface area (ECSA)**: The ECSA was determined by measuring the double-layer capacitance (C_DL_) within a specific potential window in the non-Faradaic region of 1.065–1.165 V (vs. RHE) at different scan rates (0.1 Vs^-1^ to 1 Vs^-1^).

$$i=Cdl \times\frac{\mathrm{dV}}{\mathrm{dt}}$$

The anodic and cathodic current densities at 1.115 V (vs. RHE) were plotted against the scan rates, and the slope of the graph was calculated to determine the double-layer capacitance (C_DL_).

**Turnover frequency (TOF):** The TOF was plotted against the overpotential to analyze the intrinsic properties of the electrocatalytic material. TOF of the sample was calculated by the formula,

$$TOF=\frac{\left( j\times A \right)}{n\times F\times m}$$

Where j, A, n, F, m is the current density, electrode surface area, number of electrons transferred per one molecule of the product (n = 2 for HER and 4 for OER/ORR), faradic constant (96485 Cmol^-1^) and total number of metal atoms (in mole) in the catalyst (calculated from ICP-OES analysis). When calculating “m,” we assumed that all the metal atoms in the catalyst participated in the reaction. The value of “m” was calculated based on the results obtained from the ICP-OES analysis.

$$TOF=\frac{\left( j\times1 \right)}{4\times96485\times m}$$

**Electrochemical Stability Measurements**

The electrochemical stability of the catalyst was evaluated using a 3-electrode configuration at constant current densities of 10 mAcm^-2^, 50 mAcm^-2^ and 100 mAcm^-2^. Platinum was used as the counter electrode, Hg/HgO as the reference electrode and 1M KOH as the electrolyte. The working electrode was prepared by coating a catalyst slurry (5% nafion) over a 1 cm^2^ area of a nickel-form support. Electrolyte samples (1 ml) were collected at regular intervals to quantify Fe and Ni leaching using ICP-MS measurements. After completing the stability test at 10 mAcm^-2^, the electrolyte was replaced before initiating the stability test at 50 mAcm^-2^; this procedure was repeated prior to the 100 mAcm^-2^ stability test.

**In-situ Raman Measurements**

All Raman measurements were carried out using a Renishaw inVia Reflex Spectrometer with a 532 nm laser as an excitation source and an acquisition time of 30s. In situ Raman experiments were conducted using a 3-electrode system, where carbon fiber paper loaded with the active catalyst was used as the working electrode, platinum wire as the counter electrode, and Ag/AgCl as the reference electrode in 0.1M KOH.

**Inductively coupled plasma optical emission spectrometry (ICP-OES)**

A 10 mg sample was dissolved in 5 ml of aqua regia and left overnight for complete dissolution. A 5 ml water was added to make it up to 10 ml volume. The resulting sample extract was then refluxed in a microwave. After refluxing, water was added to make up the volume to 100 ml and this was denoted as the solvent extract. The 1 ml of the diluted sample extract was used for direct analysis in ICP-OES. The equation below estimates the amount of metal in our material.

*Mass of metal atoms (/mg) = (Instrumental reading (mg/l)/1000)*(100/mass of material)*

**Inductively coupled plasma - Mass spectrometry (ICP-MS)**

A 66 µl con.HNO_3_ was added to 1 ml of the collected electrolyte to neutralize the solution. The mixture was then diluted to a total volume of 10 ml to minimize the adverse effects of the high KOH concentration. Further dilution was avoided due to the low concentrations of Fe and Ni ions, which were close to instrument detection limits.

**Computational Methodology:**

The first principles calculations were carried out within the framework of Density Functional Theory (DFT) as implemented in Vienna *Ab-initio* Simulation Package (VASP).^1-3^ We employed projector augmented wave (PAW) pseudo-potentials using of generalized gradient approximation (GGA) parametrization. ^4, 5^ The surface properties and energetics of NiFe-LDH were determined using the GGA-DFT+U.^6-8^ These calculations were conducted under the theory level of PBE+U with U_Ni_ = 4.5 eV and U_Fe_ = 3.3 eV.^9-11^ For structural optimization and electronic properties, we used 2× 5 × 1 and 3 × 6 × 1 Gamma-centered k-grid respectively for NiFe-LDH structure.^12^ The cut-off energy for a plane wave expansion was set at 500 eV. The atomic positions were optimized until forces on the atoms were below 0.01 eV/Å. Van der Waals dispersion correction were included at the DFT-D2 level.^13^ Convergence was achieved by optimizing the adsorption behavior of *O, *OH, and *OOH intermediates, with ΔG for each OER step calculated using the following equation:

ΔG = ΔE_DFT_ + ΔZPE - TΔS

In standard conditions (pH = 0, pressure, *p*_H2_ = 1 bar, and T = 298.15 K), ΔE_DFT_, ΔZPE and ΔS indicates the changes in DFT computed total energies, zero-point energy, and entropy, respectively.


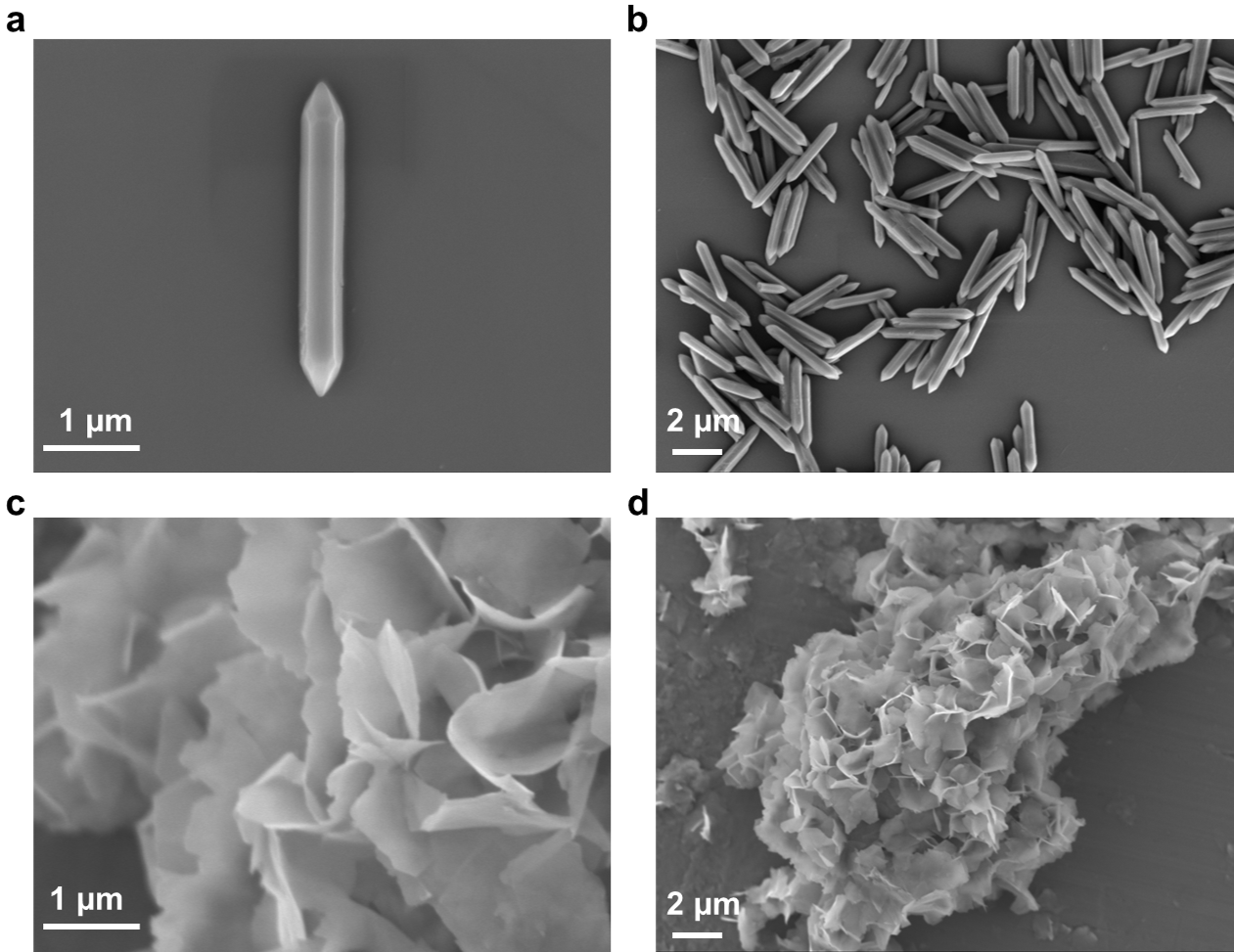


**Figure S1.** SEM images of a, b) MIL88A MOF and c & d) NiOH nano sheets.


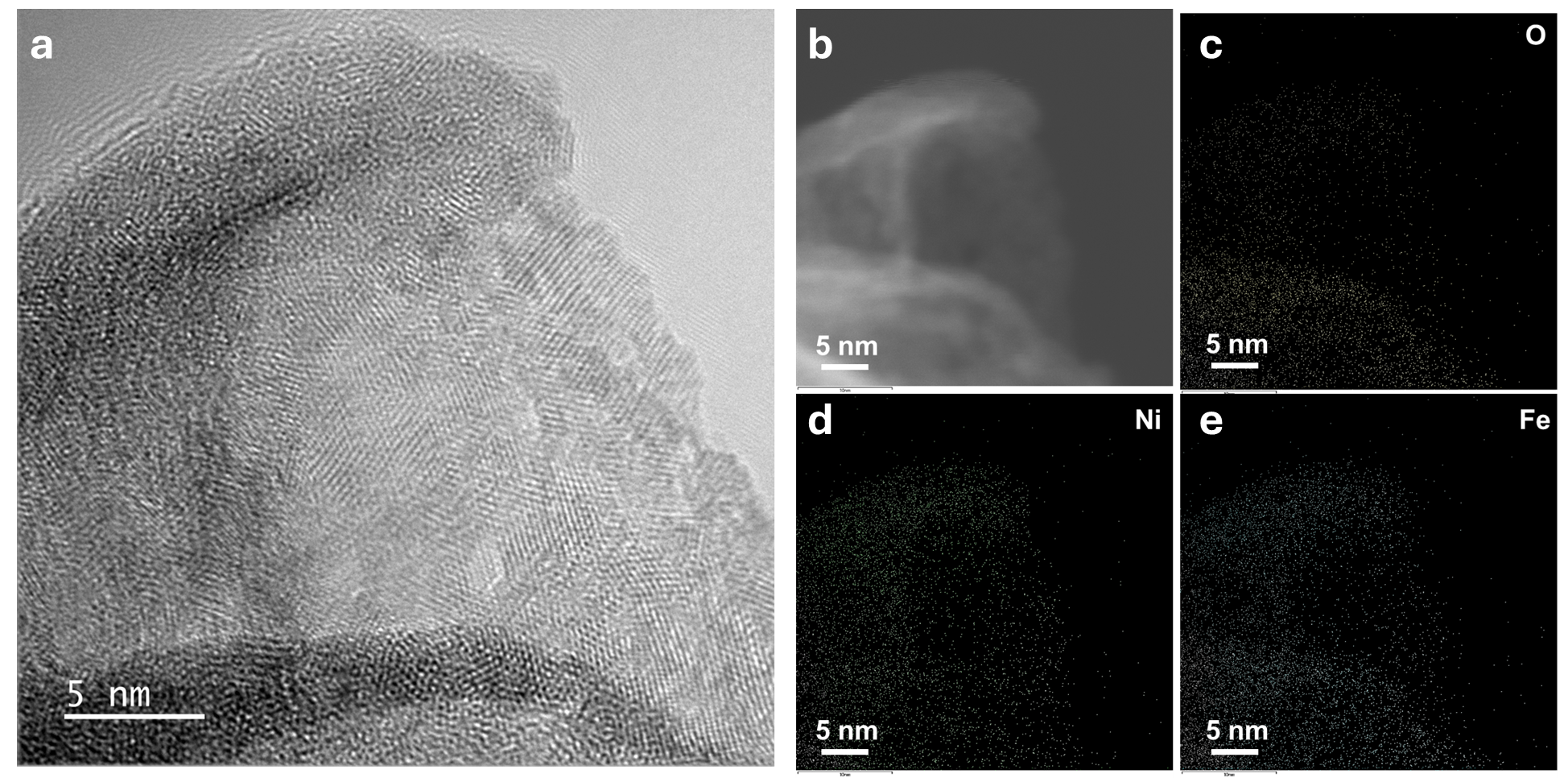


**Figure S2.** HR-TEM image and elemental mapping showing the distribution of Ni, Fe and O atoms of H-NiFe-LDH.


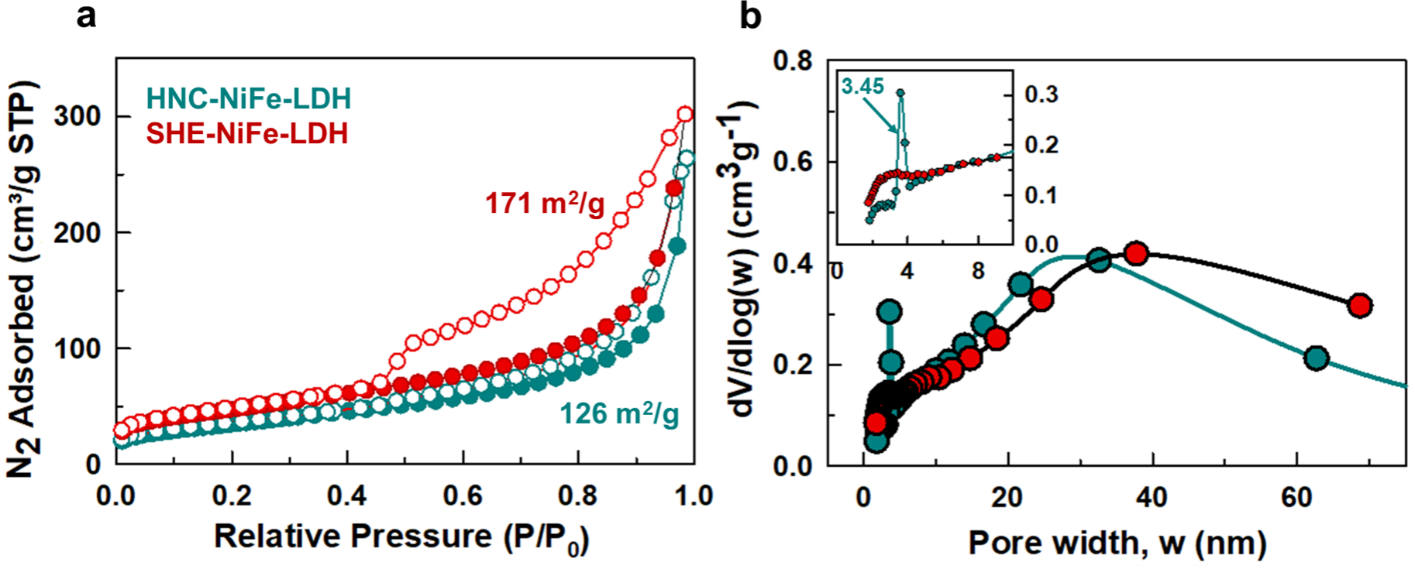


**Figure S3.** (a) N_2_ adsorption-desorption isotherms and (b) pore size distributions of H-NiFe-LDH and S-NiFe-LDH. H-NiFe-LDH (dark cyan) and S-NiFe-LDH (dark red).


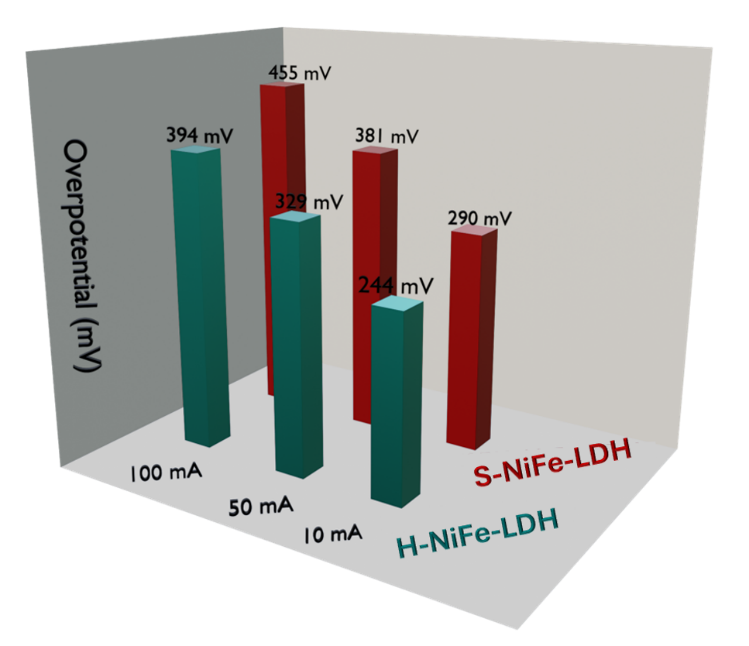


**Figure S4.** The comparison of overpotential for NiFe-LDH samples at different current densities of 10, 50 and 100 mAcm^-1^. H-NiFe-LDH (dark cyan) and S-NiFe-LDH (dark red).

**
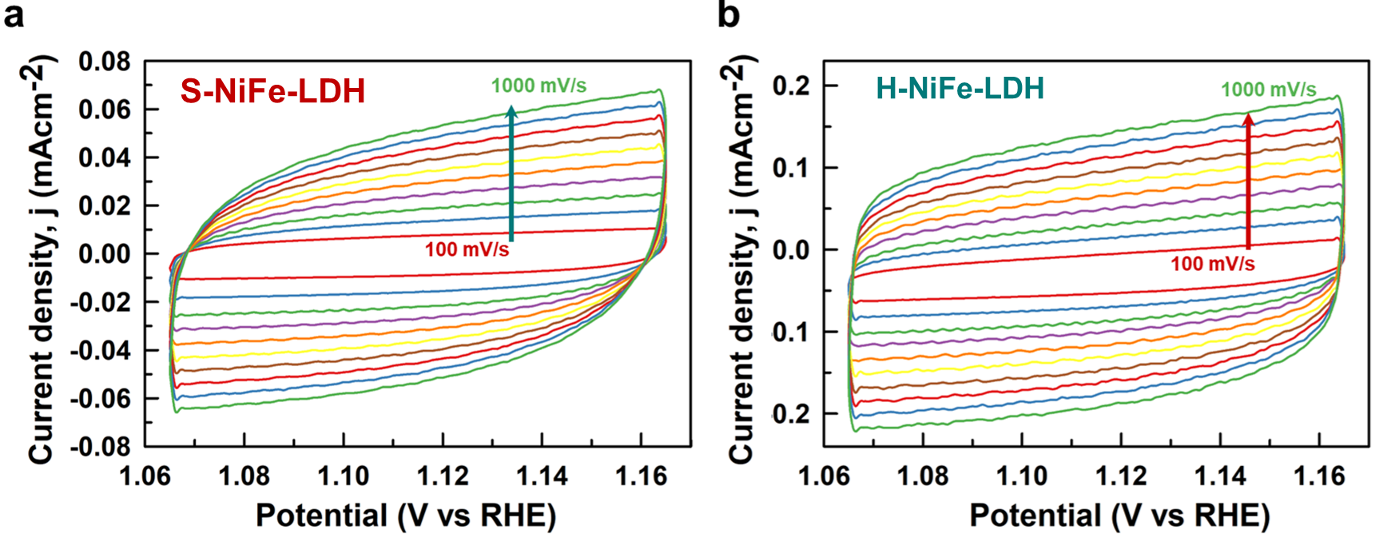
**

**Figure S5.** Cyclic voltammograms of S-NiFe-LDH and H-NiFe-LDH samples in double layer region at different current scan rates of 100 to 1000 mVs^-1^ with an increment of 100mVs^-1^ changing along the arrow direction.


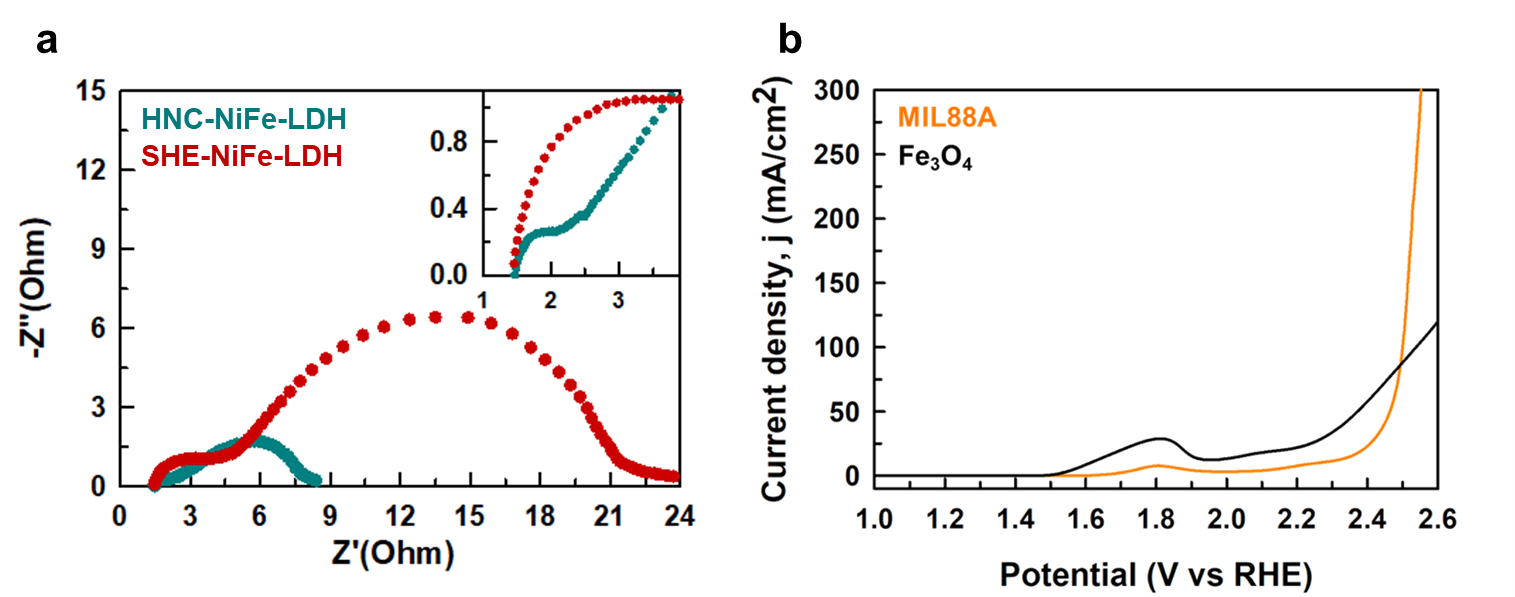


**Figure S6:** (a) Nyquist plot of H-NiFe-LDH and S-NiFe-LDH. (b) LSV curve of reference MIL88A and Fe_3_O_4_.


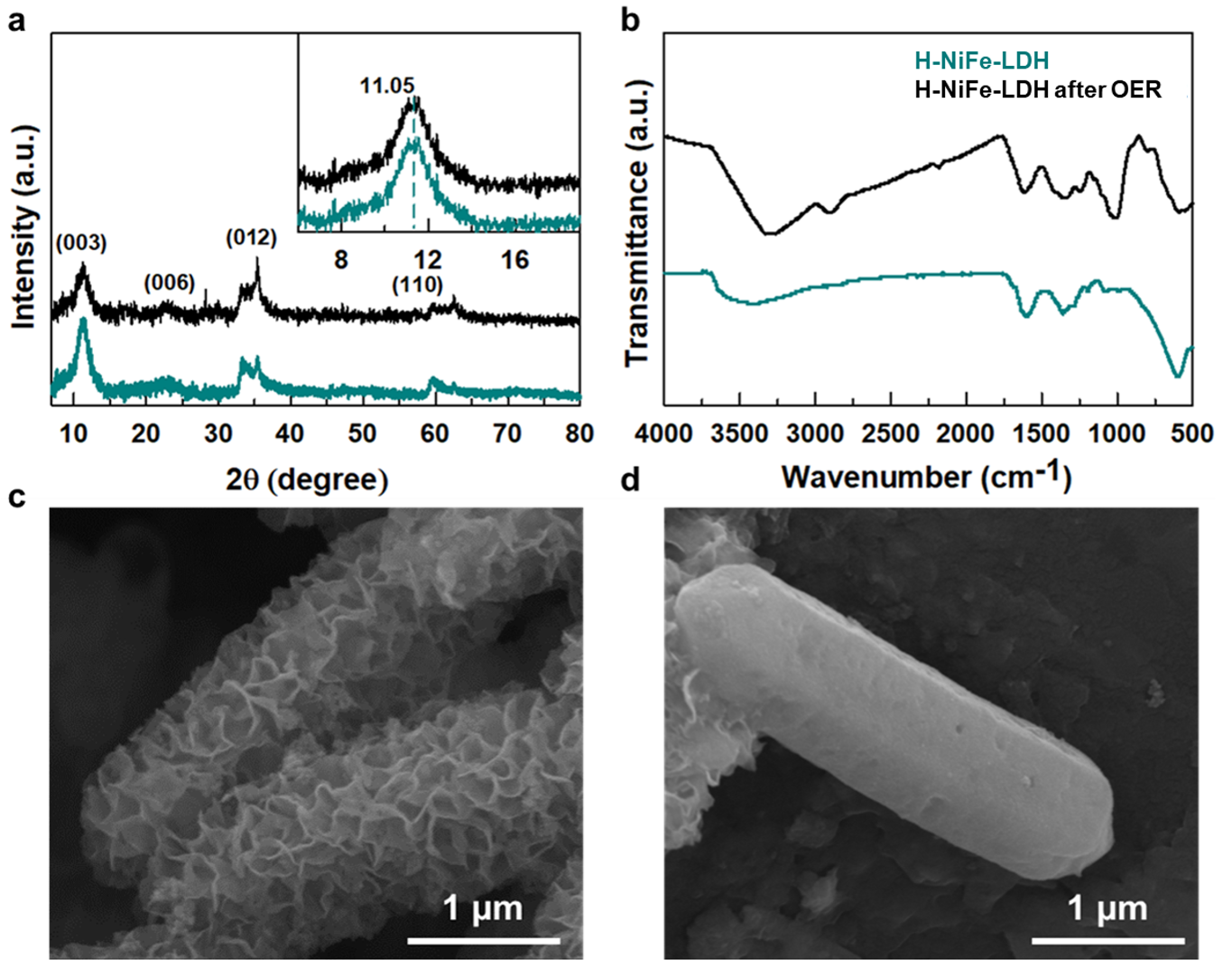


**Figure S7.** a) XRD and b) FTIR spectroscopy of H-NiFe-LDH before and after OER measurement. SEM images of c) S-NiFe-LDH and d) H-NiFe-LDH after OER measurement. H-NiFe-LDH (dark cyan) and H-NiFe-LDH after OER (black).


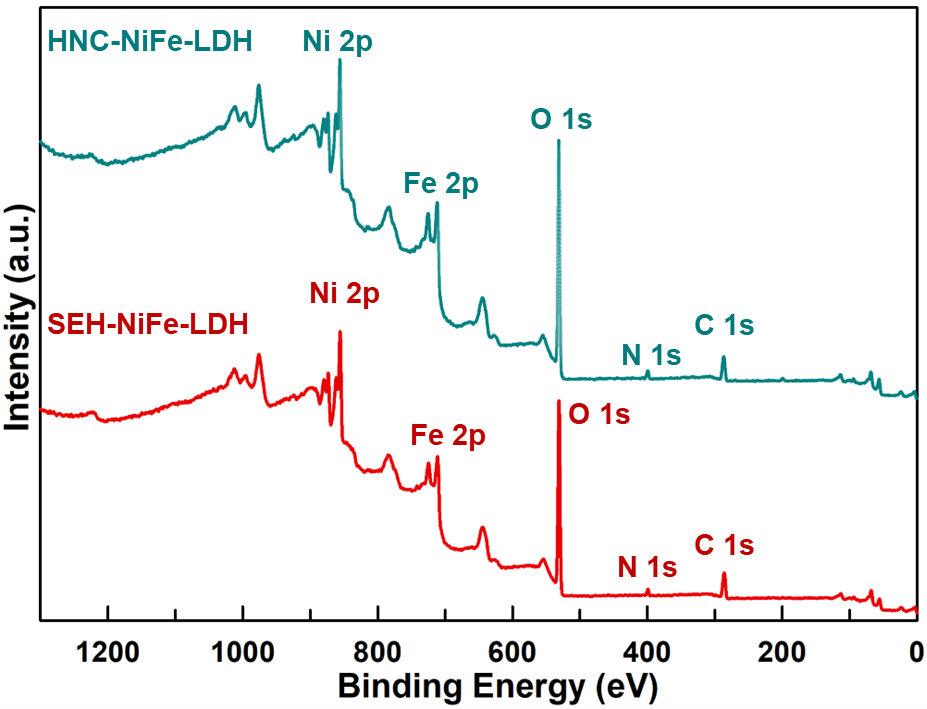


**Figure S8.** XPS survey spectra of H-NiFe-LDH and S-NiFe-LDH. H-NiFe-LDH (dark cyan) and S-NiFe-LDH (dark red).


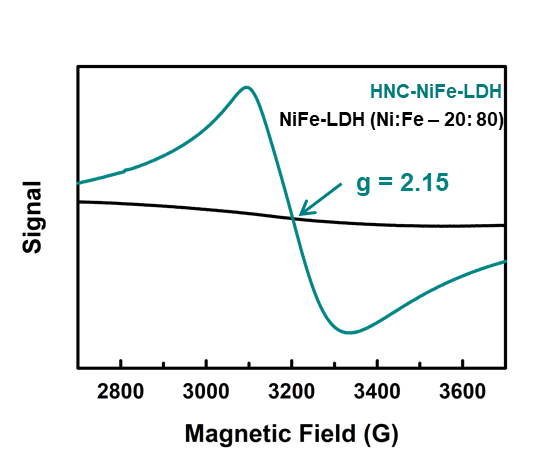


**Figure S9:** Electron Paramagnetic Resonance spectroscopy of H-NiFe-LDH and reference NiFe-LDH.

**
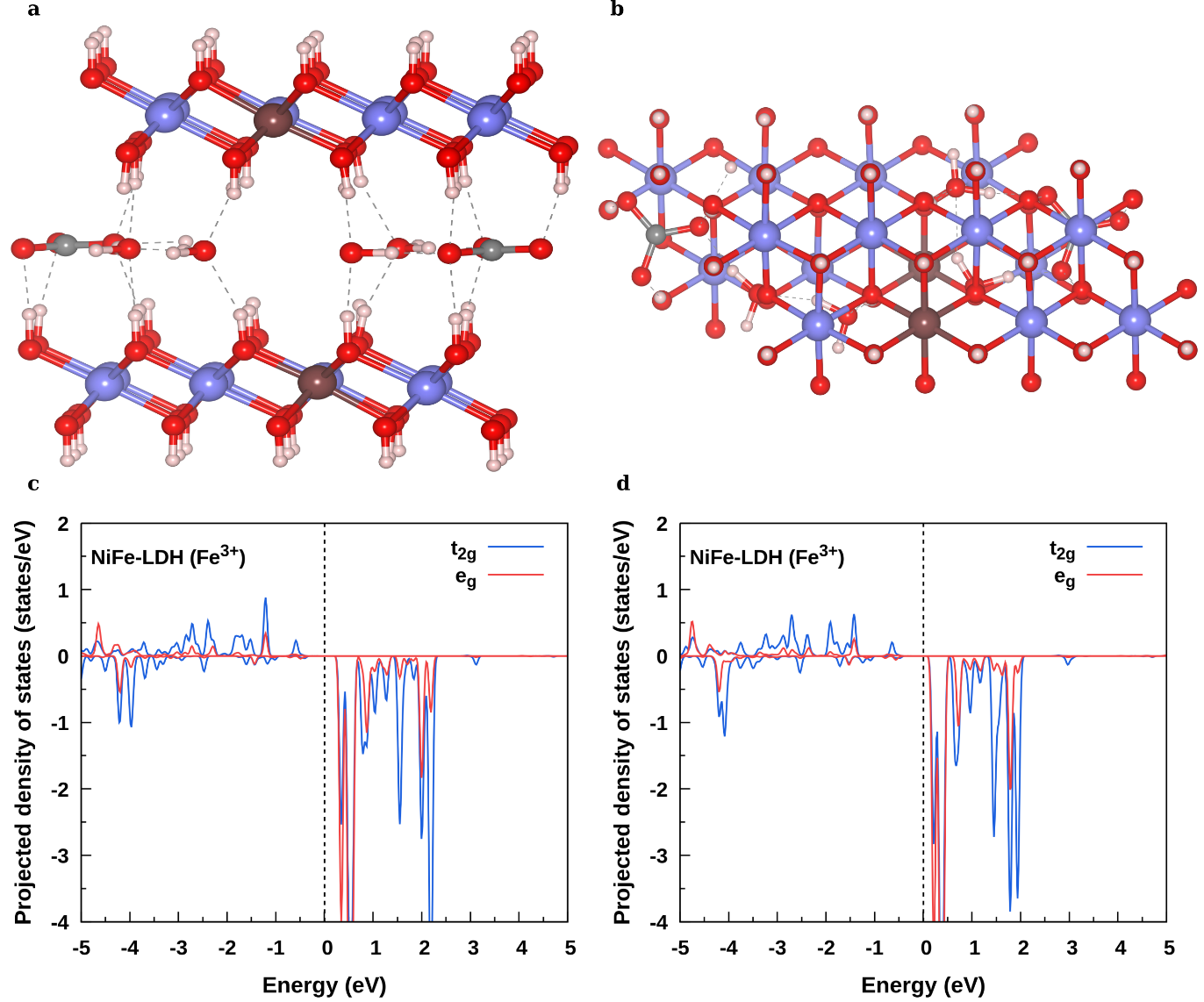
**

**Figure S10:** Models for bilayer NiFe-LDH structure a) and b) side and top view of Ni_7_Fe_1_-LDH, respectively, where brown, purple, red, grey and light pink colour denotes Fe, Ni, O, C and H atoms respectively. c) and d) Projected Density of States of Fe-d orbital for Ni_7_Fe_1_-LDH. The red and blue lines represent the e_g_ and t_2g_ orbitals and Fermi level is shifted to zero. Nickel (blue), Iron (brown), Oxygen (Red), Hydrogen (White).


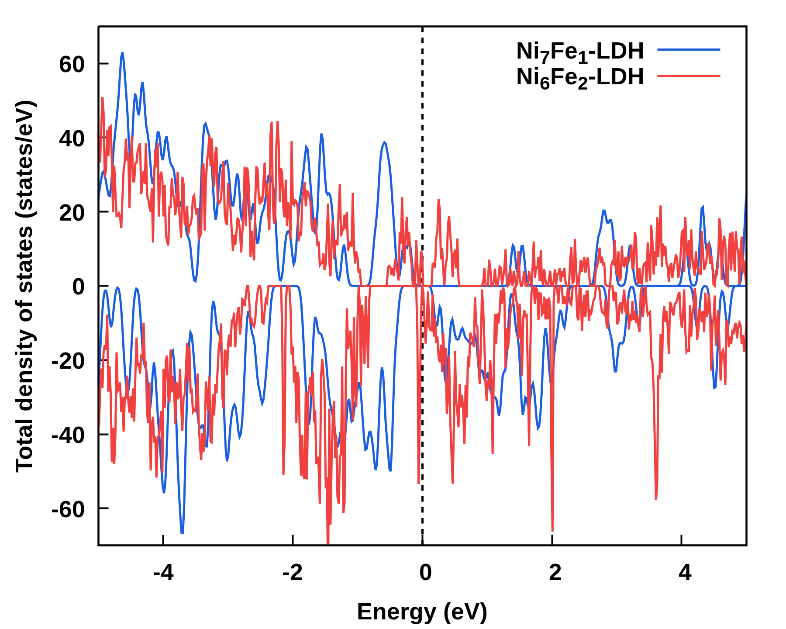


**Figure S11:** Total Density of States of for Ni_7_Fe_1_-LDH and Ni_6_Fe_2_-LDH.

**Table S1**: XPS analysis: Deconvoluted Ni 2p spectra peak area of NiFe-LDH before and after OER analysis.

|  | Ni 2p_3/2_ (Area %) | | | Ni 2p_1/2_ (Area %) | | |
| --- | --- | --- | --- | --- | --- | --- |
| Oxidation state | Ni^2+^ | Ni^3+^ | Sat. | Ni^2+^ | Ni^3+^ | Sat. |
| Binding energy (± 0.3eV) | 855.20 | 856.60 | 861.32 | 872.68 | 874.06 | 879.06 |
| H-NiFe-LDH | 26.48 | 10.28 | 34.49 | 8.88 | 7.88 | 12 |
| H-NiFe-LDH-OER | 27.67 | 8.7 | 34.8 | 10.08 | 6.51 | 12.25 |
| S-NiFe-LDH | 25.28 | 11.15 | 32.65 | 9.96 | 7.56 | 13.41 |
| S-NiFe-LDH-OER | 25.77 | 11.77 | 31.53 | 12.04 | 6.26 | 12.63 |

**Table S2:** XPS analysis: Deconvoluted Fe 2p spectra peak area of NiFe-LDH before and after OER analysis.

|  | Fe 2p_3/2_ (Area %) | | | Fe 2p_1/2_ (Area %) | | |
| --- | --- | --- | --- | --- | --- | --- |
| Oxidation state | Fe^2+^ | Fe^3+^ | Sat. | | Fe^2+^ | Fe^3+^ |
| Binding energy (± 0.3eV) | 710.55 eV | 713.97 | 718.23 | | 723.42 | 725.83 |
| H-NiFe-LDH | 47.53 | 15.24 | 11.82 | | 17.43 | 7.98 |
| H-NiFe-LDH-OER | 41.93 | 21.5 | 15.46 | | 14.76 | 6.35 |
| S-NiFe-LDH | 47.67 | 15.6 | 11.26 | | 17.13 | 8.34 |
| S-NiFe-LDH-OER | 41.01 | 23.48 | 14.06 | | 13.48 | 7.97 |

**Table S3:** XPS analysis: Deconvoluted O 1s spectra peak area of NiFe-LDH before and after OER analysis.

|  | O 1s (peak area %) | | |
| --- | --- | --- | --- |
| Oxygen contribution | O_L_ | O_v_ | Ads. H_2_O |
| Binding energy (± 0.3eV) | 529.3 | 530.7 | 532.1 |
| H-NiFe-LDH | 20.1 | 76.2 | 3.7 |
| H-NiFe-LDH-OER | 4.0 | 84.7 | 11.3 |
| S-NiFe-LDH | 22.1 | 71.4 | 6.5 |
| S-NiFe-LDH-OER | 6.6 | 86.8 | 6.6 |

**Table S4.** OER activity comparison of various catalysts in 1M KOH.

| Catalyst | Overpotential, Ƞ (10mAcm^-2^) | Tafel slope | Reference |
| --- | --- | --- | --- |
| H-NiFe-LDH | 244 | 61 mV dec^-1^ | This work |
| S-NiFe-LDH | 290 | 68 mV dec^-1^ | This work |
| Fe-doped NiV-LDH | 255 | 56 mV dec^-1^ | ^14^ |
| Fe-doped NiCoLDH | 285 mV | 62 mV dec^-1^ | ^15^ |
| NiFe-LDH/CNT@GNR | 261 | 78 mV dec^-1^ | ^16^ |
| NiFe-MOF-74 | 299 | 49 mV dec^-1^ | ^17^ |
| NiFe LDH nanosheets | 270 | 49 mV dec^-1^ | ^18^ |
| FeBi@FeNi LDH | 246 | 56 mV dec^-1^ | ^19^ |
| FeNi-LDH/Ti_3_C_2_-MXene | 298 | 43 mV dec^-1^ | ^20^ |
| FeNi hydroxide/GMC | 320 | 57 mV dec^-1^ | ^21^ |
| NiCo LDH nanosheets | 367 | 40 mV dec^-1^ | ^22^ |
| Ni-Fe LDH nanosheets. | 280 | 49 mV dec^-1^ | ^23^ |
| Ni(CN)_2_/NiSe_2_ | 270 | 68 mV dec^-1^ | ^24^ |
| Br-Ni-MOF | 306 | 79 mV dec^-1^ | ^25^ |
| NiCoFe-HO@NiCo-LDH | 278 | 50 mV dec^-1^ | ^26^ |

**Reference:**

(1) Hohenberg, P.; Kohn, W. Inhomogeneous electron gas. *Physical review* **1964**, *136* (3B), B864.

(2) Kohn, W.; Sham, L. J. Self-consistent equations including exchange and correlation effects. *Physical review* **1965**, *140* (4A), A1133.

(3) Kresse, G.; Furthmüller, J. Efficient iterative schemes for ab initio total-energy calculations using a plane-wave basis set. *Physical review B* **1996**, *54* (16), 11169.

(4) Blöchl, P. E. Projector augmented-wave method. *Physical review B* **1994**, *50* (24), 17953.

(5) Perdew, J. P.; Burke, K.; Ernzerhof, M. Generalized gradient approximation made simple. *Physical review letters* **1996**, *77* (18), 3865.

(6) Anisimov, V. I.; Aryasetiawan, F.; Lichtenstein, A. First-principles calculations of the electronic structure and spectra of strongly correlated systems: the LDA+ U method. *Journal of Physics: Condensed Matter* **1997**, *9* (4), 767.

(7) Anisimov, V. I.; Zaanen, J.; Andersen, O. K. Band theory and Mott insulators: Hubbard U instead of Stoner I. *Physical Review B* **1991**, *44* (3), 943.

(8) Bacq, O. L.; Pasturel, A.; Bengone, O. Electronic structure: Wide-band, narrow-band, and strongly correlated systems-Impact on electronic correlations on the structural stability, magnetism, and voltage of LiCoPO4 battery. *Physical Review-Section B-Condensed Matter* **2004**, *69* (24), 245107-245107.

(9) Dudarev, S. L.; Botton, G. A.; Savrasov, S. Y.; Humphreys, C.; Sutton, A. P. Electron-energy-loss spectra and the structural stability of nickel oxide: An LSDA+ U study. *Physical Review B* **1998**, *57* (3), 1505.

(10) Cococcioni, M.; De Gironcoli, S. Linear response approach to the calculation of the effective interaction parameters in the LDA+ U method. *Physical Review B—Condensed Matter and Materials Physics* **2005**, *71* (3), 035105.

(11) Zhou, Y.; López, N. The role of Fe species on NiOOH in oxygen evolution reactions. *Acs Catalysis* **2020**, *10* (11), 6254-6261.

(12) Monkhorst, H. J.; Pack, J. D. Special points for Brillouin-zone integrations. *Physical review B* **1976**, *13* (12), 5188.

(13) Grimme, S.; Antony, J.; Ehrlich, S.; Krieg, H. A consistent and accurate ab initio parametrization of density functional dispersion correction (DFT-D) for the 94 elements H-Pu. *The Journal of chemical physics* **2010**, *132* (15).

(14) Deng, Y.; Lu, Y.; Dai, R.; Xiang, M.; Zhang, Z.; Zhang, X.; Zhou, Q.; Gu, H.; Bai, J. Designing hierarchical iron doped nickel-vanadium hydroxide microsphere as an efficient electrocatalyst for oxygen evolution reaction. *Journal of Colloid and Interface Science* **2022**, *627*, 215-223.

(15) Septiani, N. L. W.; Kaneti, Y. V.; Guo, Y.; Yuliarto, B.; Jiang, X.; Ide, Y.; Nugraha, N.; Dipojono, H. K.; Yu, A.; Sugahara, Y. Holey assembly of two‐dimensional iron‐doped nickel‐cobalt layered double hydroxide nanosheets for energy conversion application. *ChemSusChem* **2020**, *13* (6), 1645-1655.

(16) Yin, X.; Hua, Y.; Hao, W.; Yang, J.; Gao, Z. Hierarchical nanocomposites of nickel/iron-layered double hydroxide ultrathin nanosheets strong-coupled with nanocarbon networks for enhanced oxygen evolution reaction. *Electrochimica Acta* **2022**, *420*, 140455. DOI: <https://doi.org/10.1016/j.electacta.2022.140455>.

(17) Rinawati, M.; Wang, Y.-X.; Chen, K.-Y.; Yeh, M.-H. Designing a spontaneously deriving NiFe-LDH from bimetallic MOF-74 as an electrocatalyst for oxygen evolution reaction in alkaline solution. *Chemical Engineering Journal* **2021**, *423*, 130204. DOI: <https://doi.org/10.1016/j.cej.2021.130204>.

(18) Suliman, M.; Al Ghamdi, A.; Baroud, T.; Drmosh, Q.; Rafatullah, M.; Yamani, Z.; Qamar, M. Growth of ultrathin nanosheets of nickel iron layered double hydroxide for the oxygen evolution reaction. *International Journal of Hydrogen Energy* **2022**, *47* (56), 23498-23507. DOI: <https://doi.org/10.1016/j.ijhydene.2022.05.147>.

(19) Wang, F.-G.; Liu, B.; Wang, H.-Y.; Lin, Z.-Y.; Dong, Y.-W.; Yu, N.; Luan, R.-N.; Chai, Y.-M.; Dong, B. Motivating borate doped FeNi layered double hydroxides by molten salt method toward efficient oxygen evolution. *Journal of Colloid and Interface Science* **2022**, *610*, 173-181. DOI: <https://doi.org/10.1016/j.jcis.2021.12.031>.

(20) Yu, M.; Zhou, S.; Wang, Z.; Zhao, J.; Qiu, J. Boosting electrocatalytic oxygen evolution by synergistically coupling layered double hydroxide with MXene. *Nano Energy* **2018**, *44*, 181-190. DOI: <https://doi.org/10.1016/j.nanoen.2017.12.003>.

(21) Wang, L.; Huang, X.; Xue, J. Graphitic mesoporous carbon loaded with iron–nickel hydroxide for superior oxygen evolution reactivity. *ChemSusChem* **2016**, *9* (14), 1835-1842.

(22) Liang, H.; Meng, F.; Cabán-Acevedo, M.; Li, L.; Forticaux, A.; Xiu, L.; Wang, Z.; Jin, S. Hydrothermal continuous flow synthesis and exfoliation of NiCo layered double hydroxide nanosheets for enhanced oxygen evolution catalysis. *Nano letters* **2015**, *15* (2), 1421-1427.

(23) Yu, L.; Yang, J. F.; Guan, B. Y.; Lu, Y.; Lou, X. W. Hierarchical hollow nanoprisms based on ultrathin Ni‐Fe layered double hydroxide nanosheets with enhanced electrocatalytic activity towards oxygen evolution. *Angewandte Chemie* **2018**, *130* (1), 178-182.

(24) Nai, J.; Xu, X.; Xie, Q.; Lu, G.; Wang, Y.; Luan, D.; Tao, X.; Lou, X. W. Construction of Ni (CN) 2/NiSe2 heterostructures by stepwise topochemical pathways for efficient electrocatalytic oxygen evolution. *Advanced Materials* **2022**, *34* (4), 2104405.

(25) Cheng, W.; Xi, S.; Wu, Z.-P.; Luan, D.; Lou, X. W. In situ activation of Br-confined Ni-based metal-organic framework hollow prisms toward efficient electrochemical oxygen evolution. *Science Advances* **2021**, *7* (46), eabk0919.

(26) Niu, Q.; Yang, M.; Luan, D.; Li, N. W.; Yu, L.; Lou, X. W. Construction of Ni‐Co‐Fe Hydr (oxy) oxide@ Ni‐Co Layered Double Hydroxide Yolk‐Shelled Microrods for Enhanced Oxygen Evolution. *Angewandte Chemie International Edition* **2022**, *61* (49), e202213049.
